# The effects of repeated lineups and delay on eyewitness identification

**DOI:** 10.1186/s41235-019-0168-1

**Published:** 2019-06-13

**Authors:** Wenbo Lin, Michael J. Strube, Henry L. Roediger

**Affiliations:** Washington University in St. Louis, One Brookings Drive, St. Louis, MO 63130-4899 USA

**Keywords:** Eyewitness identification, Confidence, Decision behaviors

## Abstract

A significant problem in eyewitness identification occurs when witnesses view a suspect in one venue such as a mugshot and then later in a lineup where the suspect is the only previously viewed person. Prior research has documented that the witness may select the suspect from the lineup due either to misplaced familiarity from seeing the mugshot or to their prior commitment from identifying the suspect from the mugshot. Two experiments attempted to minimize these biases by using repeated identical lineups, such that both targets and fillers were repeated, to determine if such a procedure could be useful. Across two experiments, we also varied the delay between seeing the event and the first lineup, as well as the delay between lineups. Despite the use of identical lineups, we continued to observe the effects of commitment and misplaced familiarity, so our procedure did not remove these problems. In addition, we also found that both repeated lineups and increasing delays can influence people’s tendency to choose and their willingness to maintain their decisions, regardless of accuracy. Most importantly, however, despite the negative effects of repeated lineups and the relatively long delays used in our experiments, we obtained strong relations between confidence and accuracy when using confidence-accuracy characteristic plots. High confidence responses were associated with high accuracy.

**Electronic supplementary material:**

The online version of this article (10.1186/s41235-019-0168-1) contains supplementary material, which is available to authorized users.

## Significance

Eyewitness misidentification in the criminal justice system is a major problem. One contributing factor is the use of repeated identification procedures, whereby witnesses’ memories are tested at two separate occasions (e.g., a photo lineup followed by a live lineup) with the suspect as the only person to appear both times. Experimental studies have shown that these procedures introduce a bias so that the suspect, even if innocent, is more likely to be selected in the second identification. The suspect may seem familiar not from having committed the crime, but merely from having been seen previously. Another problem is that witnesses tend to stay committed to their initial identification decision even if it is wrong (the commitment effect).

We developed a procedure to potentially overcome these problems by using identical lineups on two occasions. Because both the suspect and the fillers were the same in the two cases, differences in familiarity should be nullified (although not differences in commitment). However, our procedure still showed effects of misplaced familiarity and commitment. The positive news from the study is that on both identifications and across various delays, witnesses’ high confidence identifications were highly accurate. Thus, the confidence-accuracy relation seemed resistant to both repetition and to retention interval. These results add to our growing understanding of eyewitnesses’ lineup identification behaviors, as well as further documenting that, on both a first and second test that are not biased, high confidence indicates high accuracy.

## Introduction

Erroneous eyewitness identification played a role in over 70% of DNA exoneration cases. One contributor to these misidentification cases involved repeated identification procedures. Witnesses may initially see a mugshot (or a show-up) followed by a lineup, or they may see consecutive lineups (e.g. a photo lineup followed by a live lineup) – and typically the suspect is the only common face between the two. Goldstein (2019) reported about a case in New York City in which a witness identified a man, St. Clair Steward, following the city’s standard mugshot procedure. After the crime, detectives had entered broad criteria into a data base: black men between 5′8″ and 6′1″ tall who had been arrested in one of three precincts in New York. The witness leafed through the mugshots until he came to Mr. Steward’s, the 31st mugshot, and the witness identified him as the perpetrator. The detectives later placed Mr. Steward in a lineup with other men, and the witness again picked Mr. Steward. He was held in jail for two months, until DNA evidence cleared him, and the case was dropped without fanfare. Of course, without DNA evidence, Mr. Steward may have been tried and convicted. This case is hardly unique. David Lee Wiggins was convicted for the rape of a 14-year-old girl in Fort Worth, Texas. The victim identified him from a photo lineup and live lineups in which Wiggins was the only repeated member in the lineups. He was later exonerated due to DNA evidence. In addition, the famous case of Ronald Cotton (wrongly convicted) and Jennifer Thompson (the victim and witness) also involved repeated lineups in which Cotton was the only repeated person. The perpetrator, Bobby Poole (as later determined by DNA evidence), was in neither lineup, but Thompson identified Cotton in both lineups, although on the first occasion it took her 5 min to do so after narrowing it down to two men.

The common denominator of these cases is that the suspect was the only repeated member in these repeated lineup procedures (single-target-repeated lineups), and studies have shown that such procedures inadvertently bias witnesses toward choosing the suspect. Prior research has focused primarily on repeated identification procedures involving a single repeated member (a guilty or innocent suspect) across multiple identifications (e.g., Godfrey & Clark, 2010; Steblay & Dysart, 2016; Steblay, Tix, & Benson, 2013); all studies showed a bias toward choosing the repeated person on the second occasion. We review the relevant research below, before suggesting a procedure that might mitigate these problems.

A second issue to be considered in our research is that of delay between witnessing an event and being examined on it, as well as the delay between the first and second opportunity to identify the suspect. Of course, longer delays are expected to lead to lower performance and more errors in eyewitness identification. We review the evidence about delay, too, before introducing our experiments that examine both the initial delay between witnessing a crime and a first identification (ID), as well as the subsequent delay between the first and second ID.

The third issue of concern in the present research is measuring the influence of repeated identifications on the confidence-accuracy (CA) relationship as measured by confidence-accuracy characteristic (CAC) plots. Despite problems with repeated identifications, will high confidence responses still indicate high accuracy, as found in virtually all studies in eyewitness memory when assessed by CAC plots on a single unbiased test (Wixted, Mickes, Clark, Gronlund, & Roediger, 2015; Wixted & Wells, 2017)?

## Problems with repeated identification procedures

Repeated identification procedures are relatively routine (Behrman & Davey, 2001; Steblay, 2011). On the surface, these procedures may appear beneficial to criminal investigations (e.g., allowing for a confirmation of an eyewitness identification), but laboratory research has consistently shown the negative effects of repeated identifications: mugshots followed by lineup (Brigham & Cairns, 1988; Deffenbacher, Bornstein, & Penrod, 2006; Memon, Hope, Bartlett, & Bull, 2002), prior show-up (seeing a person) followed by a lineup (Godfrey & Clark, 2010; Valentine, Davis, Memon, & Roberts, 2012) and consecutive lineups (Hinz & Pezdek, 2001; Pezdek & Blandon-Gitlin, 2005; Steblay & Dysart, 2016; Steblay et al., 2013). In a show-up usually one person is viewed whereas with mugshots, witnesses often see several or more (31 in the case mentioned above). Unlike in mugshots or show-ups, the suspect in a lineup is presented alongside fillers, which offers some protection against identification errors in that the witness may pick a person known to be innocent or no one at all. Nonetheless, Steblay et al. (2013) still showed that identification errors from the initial lineup were often carried over to the subsequent lineup when the suspect was the only person in both lineups. Similarly, Hinz and Pezdek (2001) found that exposure to an innocent suspect in one target-absent lineup increased the false alarm rate to that innocent person if the same person appeared in a subsequent target-absent lineup. This presumably occurred because only one face was familiar in the second lineup, and this familiarity was misattributed to having seen the person commit the crime rather than from the person appearing in the previous lineup. Thus, the mechanism at work – misattribution of familiarity – may be the same when two successive lineups are viewed with only one person in common, as in the studies when witnesses see a show-up or mugshot and then are given a lineup. In both cases, an innocent person may be selected due to misattribution of familiarity, as occurs in other studies, too (see Jacoby, Kelley, & Dywan, 1989).

Hinz and Pezdek (2001) observed that exposure to an innocent filler in a first target-absent lineup also hurt performance when the second lineup was a target-present lineup (i.e., it included the suspect). The repeated filler (among other fillers) reduced the hit rate of a subsequent target-present lineup (.42) compared to when the guilty suspect was presented with all new fillers (.76). Together, these studies demonstrated the negative effects of repeated lineups, but their findings reflected procedures where witnesses may have been biased towards choosing the single-repeated target. Our experiments attempt to mitigate this problem by using the same lineup on two occasions, for reasons discussed below.

We have thus far emphasized misattributions of familiarity as a root cause of the problem with repeated IDs with only one person repeated, but at least three different problems potentially exist. Besides misplaced familiarity, two other possible problems are misinterpretation of the police’s intention and witnesses’ commitment to a prior identification decision. For example, when a particular face is repeated across lineups, witnesses may unintentionally misinterpret the police’s intention and believe that the police have identified that particular lineup member as the perpetrator. In addition, witnesses may select an innocent suspect in both the initial and subsequent identification to remain consistent with their initial decision (a commitment effect). The commitment effect has been observed in the misinformation literature (e.g., Schreiber & Sergent, 1998); however, it also applies to the eyewitness identification literature. For example, Valentine et al. (2012) reported that most choosers (88%) in a show-up also selected the same suspect in the subsequent lineup, regardless of whether the initial identification was accurate. This commitment effect has also been observed in other studies (Dysart, Lindsay, Hammond, & Dupuis, 2001; Godfrey & Clark, 2010; Haw, Dickinson, & Meissner, 2007). Eyewitnesses may perceive inconsistent decisions across identifications as an indication of unreliability; therefore, they might feel compelled to stick with their initial decision.

Given these findings, one target for research is whether changes in dual identification procedures could mitigate or eliminate these effects. If both the suspect and fillers are repeated in two lineups, all members would be familiar. Thus, problems due to misplaced familiarity or to misunderstanding what the police (or experimenter) are seeking should, theoretically, be eliminated. Although repeating identical lineups would not necessarily remove the commitment effect, it should yield a better measure of the commitment effect because it would be potentially uncontaminated by the other two issues.

One possible positive use of repeated identical lineups is that performance (identifying the suspect) may improve. Improvement in recall or recognition between two tests is called hypermnesia, and it has been reported in free recall tasks quite regularly (e.g., Erdelyi & Becker, 1974; Roediger & Payne, 1982). Recognition hypermnesia has been reported by Bergstein and Erdelyi (2008), although Payne and Roediger (1987) did not find it with word-list stimuli. If witnesses receive an additional identification opportunity (a subsequent unbiased lineup), would they show hypermnesia? The use of identical lineups may eliminate problems with single-repeated-target lineups and permit greater accuracy on a second test than a first test.

## The effects of delay on repeated lineups

The retention interval (or delay) between witnessing an event and being examined on it is a critical variable in all memory research, and eyewitness identification is no exception. However, in repeated identification procedures, there are two relevant delays: the delay between the crime and the initial lineup (the initial delay) and the delay between two repeated identifications (we will refer to this as the subsequent delay).

Numerous researchers have examined how the initial delay affects eyewitness identification, but the effects vary across studies. A longer delay has been shown to decrease the number of correct identifications in some studies (Cutler, Penrod, O’Rourke, & Martens, 1986; Sauer, Brewer, Zweck, & Weber, 2010); however, Egan and colleagues (1977) manipulated retention interval across 2, 21 and 56 days and observed no decrease in correct identifications, but they did observe an increase in false identifications (hence overall accuracy declined). Valentine, Pickering, and Darling (2003) observed a 65% suspect identification rate on an immediate test, but they did not find a reliable decrease in suspect identification rate when the test occurred after 1 or 6 months, although performance was lower (the suspect ID rate fluctuated between 34 and 46%). These null results may have been due to low statistical power. Whereas the effects of delay on identification are not perfectly consistent across studies, they generally suggest poorer identification performance with an increase in delay, confirming over a century of memory research using other procedures (e.g., Nairne, 2008). Importantly, though, studies that have examined the confidence-accuracy relationship with varying delays show that it holds up well across delays as assessed by CAC plots. That is, even though witnesses identify fewer suspects after long delays, if they are highly confident in their identifications, they are generally highly accurate (Palmer, Brewer, Weber, & Nagesh, 2013; Sauer et al., 2010; Wixted, Read, & Lindsay, 2016). However, these studies examined the effects of the retention interval between witnessing a crime and the first identification; no research has examined the effects of the subsequent delay between two lineup identifications on the confidence-accuracy relationship.

As just noted, unlike the initial delay, the effects of the subsequent delay in repeated identifications has received far less attention. The subsequent delay may influence how eyewitnesses make their second identification decisions and their tendency to switch choices. For example, Godfrey and Clark (2010) found that when the delay between a first and second identification was short (30 min), witnesses generally made consistent decisions (e.g. selecting the same person in both identifications). However, when the delay was one week, they found that witnesses made a combination of consistent decisions and switched decisions (from not picking a suspect the first time to picking one on the second occasion, the no-to-suspect shift). The long delay increased the proportion of no-to-suspect shifts. Steblay et al. (2013) used a 2-week delay between two (non-identical) lineups and they also found a mixture of consistent decisions and no-to-suspect shifts. Although the length of subsequent delay appears to influence witness identification behaviors, no researchers have yet examined CAC plots to see how the confidence-accuracy relationship changes with repeated tests.

## Repeated identification and the confidence-accuracy relationship

In addition to reducing suggestibility in repeated lineups and examining the effects of delay, another critical issue is whether the CA relation remains strong across repeated IDs. Eyewitness confidence has been widely used and endorsed as an indicator of accuracy by the U.S. Supreme Court, police, and jurors (Brewer & Burke, 2002; Deffenbacher & Loftus, 1982; Potter & Brewer, 1999). The CA relationship in eyewitness identification has been studied extensively (Bothwell, Deffenbacher, & Brigham, 1987; Juslin, Olsson, & Winman, 1996; Sporer, Penrod, Cutler, & Read, 1995). Through the use of CAC plots, the current state of the evidence indicates that high confidence in an initial lineup indicates high accuracy (Wixted et al., 2015). As noted above, several studies have shown that retention interval does not undermine the CA relationship (Palmer et al., 2013; Sauer et al., 2010; Wixted et al., 2016). However, these studies used single tests, and our research will ask if the relation is maintained on the second lineup when repeated lineups are used.

Steblay and Dysart (2016) recommended against the use of repeated identifications involving the same suspect, but it is unclear from their recommendations whether repeated identifications significantly impair the CA relationship measured using the CAC plots (Wixted & Wells, 2017). There are at least two ways that repeated identifications can affect the CA relation. For instance, witnesses who initially had low confidence in their identification decision may become more confident in a subsequent identification (confidence inflation). Prior research has shown that confidence inflation can occur if witnesses had previously received confirmatory feedback (Bradfield & Mcquiston, 2004; Wells & Bradfield, 1998; Wells, Olson, & Charman, 2003); however, it is uncertain whether repeating testing alone (e.g., repeated identification procedures) causes confidence inflation. Godfrey and Clark (2010, Experiment 2) found that people who consistently chose the suspect were more confident in the subsequent lineup than in the initial show-up, but Steblay et al. (2013) did not find a confidence inflation effect when people consistently chose the suspect in both an initial and a subsequent lineup with only the suspect in common. Alternatively, witnesses may maintain the same level of confidence across lineups (Steblay et al., 2013), perhaps remembering their level of confidence from the first occasion (a confidence carryover effect). Furthermore, the level of confidence expressed at an earlier identification may not only carryover to a subsequent identification, but it may also predict the commitment effect (e.g., high confidence individuals are more likely to commit to their initial decisions than low confidence individuals). In short, confidence inflation could produce poorer confidence and accuracy calibration (e.g., if identification errors are made with higher confidence in Lineup 2 than Lineup 1), but a confidence carryover effect could maintain the CA relationship if it exists in the first lineup. Because no research has examined the effects of repeated lineups (in our experiments, identical lineups) on the CA relationship using CAC plots, our experiments are the first to address this issue.

## The present study

The follow questions summarize the objectives of the present study: 1) What are the effects of repeated identical lineups and delay on the tendency to choose from a lineup and on identification accuracy? 2) Given an unbiased subsequent lineup (an identical lineup), are witnesses still committed to their initial decision? 3) Does confidence inflate with repeated lineups? Or does it produce a confidence carryover effect? 4) More importantly, does the confidence-accuracy relationship remain intact despite the effects of repeated lineups and varying delays between them? Because prior studies have shown that both repeated lineups and delay can harm identification accuracy, the primary issue addressed in the current is experiments is whether the combination of these variables significantly impairs the confidence-accuracy relationship.

We report two experiments that investigated the joint effects of repeated (identical) lineups and retention interval on eyewitness identification accuracy and the confidence-accuracy relation. The use of repeated lineups was designed to reduce the problems of selective familiarity of the suspect and the demand characteristic of having only one suspect repeated. Such lineups may be difficult but not impossible to carry out in police departments (e.g. photo lineups could be used on both occasions, as in our experiments). We also varied the delays between the event and the first lineup (initial delay), as well as between the two lineups (subsequent delay), to determine effects on identification performance and the CA relationship (measured by CAC plots). Manipulating both the initial interval and the subsequent interval permits us, in certain conditions, to examine the effect of repeated testing without a confound of delay (i.e., the second test can be given in one condition at the same retention interval as the initial test in a different condition). In short, repeated lineups and delay tend to co-occur in the real world, and prior research has often only manipulated one of these two variables but not both.

We predicted a greater tendency to choose from the second lineup than the first lineup, both because all faces were repeated (enhancing their general familiarity). That is, we expected to observe nonchooser-to-chooser shifts based on familiarity of the faces, but familiarity would not accrue only to the suspect in target-present lineups, as it has in previous research. On the other hand, we should observe a greater tendency to choose after a short-initial delay than a long-initial delay because people may feel less confident in their ability to make an identification after a long delay due to forgetting. Identification accuracy (the number of guilty suspect IDs) should decrease with an increase in retention interval, and we expect more identification errors in the subsequent lineup than the initial lineup due to misattributions of familiarity accruing to all lineup members. There is no evidence from prior repeated lineups studies about whether the CA relationship will remain intact in a repeated lineup; however, based on prior literature, confidence inflation and confidence carryover effects will probably exist and could potentially harm the CA relationship. The present study also compared confidence judgments and response time in making judgments as indicators of accuracy and found confidence judgments were more predictive of accuracy than response times (see the Additional file 1 for the response time data).

In short, we expected our experiments to confirm some findings from the prior literature and to address several issues for the first time regarding repeated lineups with the same fillers on both occasions. We also have a larger sample size and hence greater numbers of observations and more power than in prior repeated lineup studies.

We will discuss Experiments 1 and 2 jointly because the same stimuli were used, and their overall designs were similar. The main difference between Experiments 1 and 2 was the length of delay between Lineup 1 and Lineup 2 (i.e., the subsequent delay). In Experiment 1, the subsequent delay was always three days. In Experiment 2, the subsequent delay was either one day (a short subsequent delay) or five days (a longer subsequent delay).

## Method

### Participants

**Fig. 1:**
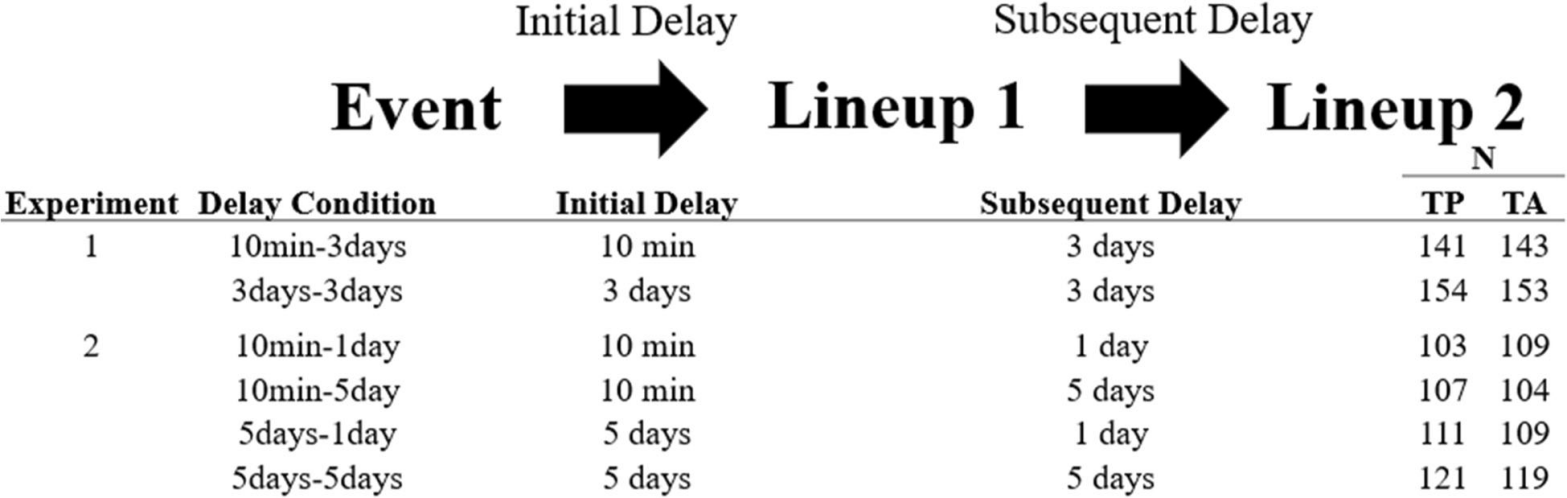
The overall design of Experiments 1 and 2. The initial delay determined the length of delay between the Event and Lineup 1, and the subsequent delay determines the length of the delay between Lineup 1 and Lineup 2. All the participants saw the same lineup two times (Lineup 1 and Lineup 2). For each delay condition, the number of participants in target-present (TP) and target-absent (TA) lineup conditions is shown in the right

For Experiment 1, 787 participants were recruited via Amazon Mechanical Turk (MTurk), but only 591 participants completed all the sessions. The remaining 196 participants consisted of those who had technical difficulties, completed the follow-up session later than the deadline, or dropped out of the study; their data were not included in the analyses. The number of participants in between-subject conditions ranged from 141 to 154 (see Fig. 1). For Experiment 2, 1158 participants were recruited via MTurk. Only 883 of them completed all the follow-up sessions. The remaining 275 participants whose data were dropped consisted of those who had technical difficulties, completed the follow-up session later than the deadline, or dropped out of the study. The number of participants in between-subject conditions ranged from 103 to 121. All participants were compensated with payment ($2.50).

### Design of Experiments 1 and 2

The design of Experiments 1 and 2 were generally similar, as shown in Fig. 1. The procedure always consisted of the event (i.e., the crime video), lineup 1 and lineup 2. In Experiment 1, the initial delay (the delay between the event and lineup 1) was either 10 min or 3 days and the subsequent delay (the delay between lineups) was always 3 days. In Experiment 2, the initial delay was either 10 min or 5 days and the subsequent delay was either 1 day or 5 days. Experiment 1 sought to replicate the effects of initial retention interval on accuracy of choices and on the CA relation. The purpose of the various intervals between lineups (1, 3 or 5 days across the experiments) was to see whether the length of the subsequent delay affected the likelihood of repeated decisions and switched decisions, as well as the CA relationship. Because the subsequent delay was held constant in Experiment 1, we did not expect to find ID differences based on the initial delay conditions (unless ID on initial delay interacts with ID in the subsequent delay). In Experiment 2, we predicted the likelihood of switched decisions to increase as a function of the subsequent delay, but the critical issue is whether the CA relation would also be undermined. We used 1-day and 5-day delays in Experiment 2 to increase the disparity in identification accuracy and (probably) the number of repeated consistent decisions. In sum, there were two delay conditions in Experiment 1 and four delay conditions in Experiment 2 (see Fig. 1). Roughly half of the participants received a target-present lineup and half received a target-absent lineup in both experiments (see Fig. 1 for the exact numbers).

In addition to the eyewitness task, participants in both experiments also completed two distractors tasks (a free recall task and a lexical decision task, described below) after seeing the crime video. Together, the two tasks took approximately 10 min. The purposes were to provide a distractor task and, if needed, to provide co-variates for later analyses. However, we did not use them for this latter purpose. In Experiment 1, participants in the 10 min initial delay condition (i.e. the 10 min-3 days condition) saw lineup 1 after the filler tasks. Those in the 3 days–3 days condition did not see lineup 1 until three days later. Similarly, in Experiment 2, participants in the 10 min-1 day and the 10 min-5 days condition saw lineup 1 after the filler tasks. Those in the 5 days-1 day and the 5 days–5 days condition did not see lineup 1 until five days later.

All sessions took place online. Email reminders were sent for each subsequent session along with a link to the online experiment and participant login information. Once they received the email, participants in Experiment 1 were given 36 h to complete the follow-up session and those in Experiment 2 were given 48 h to complete the follow-up session. The longer time window in Experiment 2 was used to minimize the number of late responses.

### Materials

The present study used the video and lineup materials from Mickes, Flowe, and Wixted (2012). The video showed a white male (the perpetrator) walking into an unoccupied office and stealing a laptop from the office desk. Viewers had a clear view of the perpetrator as he left the office with the laptop. The video lasted for 23 s and the perpetrator’s face was in view for roughly 4–5 s.

Participants received either a target-present or target-absent lineup consisting of six people. The only difference between the two lineups was that the target in the target-present lineup was replaced by a filler in the target-absent lineup. The same lineup was used twice, but the position of the photos was randomized on both occasions. The lineup photos from Mickes et al. (2012) were digitally modified so that all the lineup members wore a black shirt. This step was taken to prevent participants from using the color of clothing as a cue. We also adjusted for the spacing between the top of the lineup member’s head and the top edge of the photo. Furthermore, because these photos were taken with different cameras, we made digital adjustments to equate the photo quality. This was to ensure that none of the photos stood out. These changes doubtless made identification in our lineups more difficult than in Mickes et al. (2012). The photos appear in the Additional file 1.

The eyewitness task materials for Experiment 2 were the same as those used in Experiment 1 except another filler in the target-absent lineup was randomly selected to replace one filler in the target-present lineup. In other words, Experiments 1 and 2 had four overlapping fillers in the target-present lineup condition. This was to see whether using another target-replacement filler would produce different frequencies in the overall filler selections.

The categorized words for the free recall task were drawn from the norms of Van Overschelde, Rawson, and Dunlosky (2004). Fifteen words from the *fruit* and the *vegetable* categories were used for the list (Van Overschelde et al., 2004). The lexical decision task consisted of 16 practice trials (4 high- and 4 low-frequency words, and 8 nonwords) and 80 experimental trials (20 high- and 20 low-frequency words, and 40 nonwords). Both the words and nonwords were taken from Yap, Balota, Tse, and Besner (2008). None of the words from the free recall task overlapped with those in the lexical decision task.

### Procedure

After general instructions about the experiment, participants saw a video of the target committing a crime. They were simply instructed to pay attention to the video. Immediately after the video, participants were asked to complete the two distractor tasks: studying and recalling words (about 5 min) and the lexical decision task (another 5 min). In the free recall task, they saw a total of 30 words in a random order for two seconds each, with a 500 ms interstimulus interval. After the last word, participants were asked to recall as many words as possible, in any order, for four minutes. Then the screen automatically advanced to the lexical decision task in which participants were given instructions to decide whether each upcoming letter string represented a word or nonword. There were 16 practice trials followed by 80 experimental trials. Each trial consisted of the following sequence of events: 1) a fixation point (+) presented at the center of the screen for 400 ms, 2) a blank screen for 200 ms. 3) appearance of the stimulus at the fixation point until the participant made a response. Participants were instructed to press the “M” key for words and the “Z” key for nonwords. If they made a correct response, a blank screen was presented for 1600 ms before the fixation point appeared again. For incorrect responses, the word “Incorrect” was shown slightly below the fixation point for 1600 ms. These tasks took at least ten minutes to complete, but the exact duration depended on the participant’s pace during the lexical decision task.

In Experiment 1, after the lexical decision task, participants saw lineup 1 in the 10 min-3 days delay condition or were asked to wait for a follow-up email in the next few days (the 3 days–3 days delay condition). Prior to seeing the lineup, participants were given instructions and were told that the suspect may or may not be present in the lineup. They made their identification decision by clicking on the face of the target, and then they clicked the “next” button to submit their choice. If participants judged that the target was not in the lineup, they could reject the lineup via the “not present” button. They were presented with either a target-present or target-absent lineup, and the same lineup was given in lineup 2 (e.g., participants saw the same target-present lineup or target absent lineup for both sessions). The position of the lineup members was randomized in both lineups. Response timing for responding started when participants saw the lineup and ended when they clicked the “Next” button after selecting one of the faces or the “not present” button. Participants were not given any instructions about how fast they should make a decision. After making their identification decision, they were asked to make a confidence judgment using a 0 to 100 scale (0 = Not confident at all; 100 = Very confident). Participants rated their confidence by moving a slider from 0, where the cursor started, to a point on the scale between that value and 100. As they moved the slider, the corresponding numerical value was displayed above the slider. Both lineups used the same procedure, and no feedback was provided about whether participants made the correct choice. At the start of their follow-up session for Lineup 2, they were asked to identify the suspect from the video and were again told that the suspect may or may not be in the lineup. After making their identification, they were asked to provide a confidence judgment using the same scale. At the end of Lineup 2, participants were thanked for their participation and debriefed.

The general lineup identification process was similar for Experiment 2, but unlike in Experiment 1, choosers in Experiment 2 did not have to click on a lineup member’s photo and the “Next” button to submit their response. That is, Experiment 1 participants were free to change their decisions before they submitted their final response. In Experiment 2, they were informed that only their first response would be recorded. This was to equate the response time between choosers and nonchoosers, because nonchoosers in both experiments only had to click on the “Not Present” button to submit their response. The confidence judgment section of the experiment was identical in both experiments.

## Results and discussion

The results that answer our basic questions are rather numerous and complex. We have divided them into sections that address the issues we intended to address, and we discuss the implications of the results after they are presented.

### Repeated identical lineups increased the tendency to choose

**Fig. 2:**
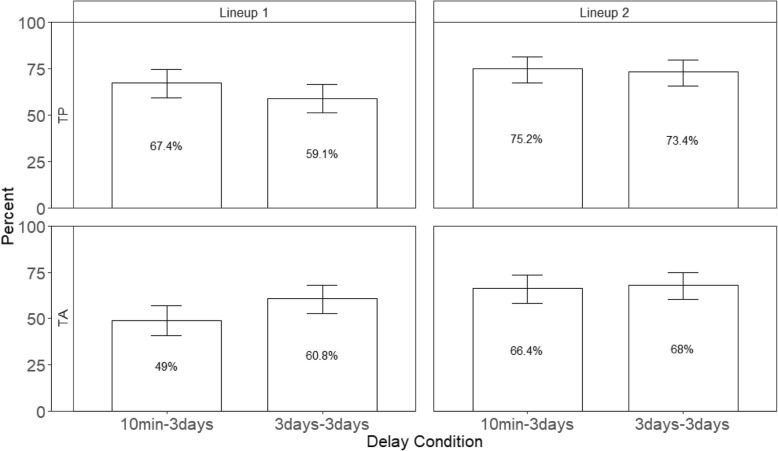
The percentage of choosers across lineups and delay conditions in Experiment 1. The top panels show the target-present lineups and the bottom panels show the target-absent lineups. Error bars represent 95% confidence intervals

**Fig. 3:**
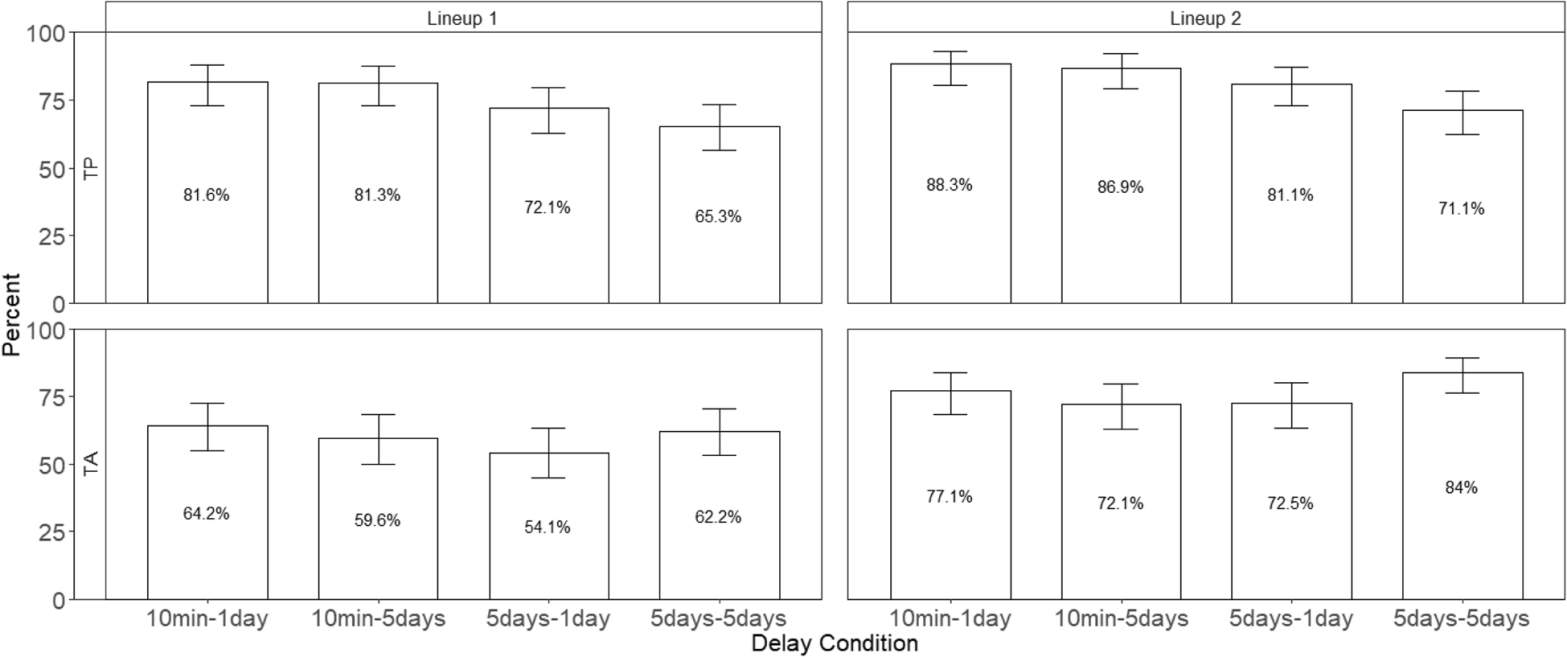
The percentage of choosers across lineups and delay conditions in Experiment 2. The top panels show the target-present lineups and the bottom panels show the target-absent lineups. Error bars represent 95% confidence intervals

Using an identical lineup procedure that made all faces somewhat familiar on the second ID resulted in an increased tendency to choose during the second lineup, just as occurs in other situations. The choosing rate (the percentage of times participants identified a face, whether right or wrong) is shown in Fig. 2 for the conditions of Experiment 1; the corresponding data for all conditions of Experiment 2 is in Fig. 3. The choosing rate for target-present lineups is presented in the top panels, whereas the choosing rate for target-absent lineups is in the bottom panels.

To evaluate our hypotheses, we used the lm4 package in R (Bates, Mächler, Bolker, & Walker, 2015; R Core Team, 2017) to conduct a 2 (repeated lineups) × 2 (delay conditions) × 2 (lineup type) generalized mixed model for Experiment 1. Although there were four delay conditions in Experiment 2, two conditions had an initial delay of 10 min and the other two conditions had an initial delay of 5 days. Therefore, we compared the two 10-min conditions with the two 5-days conditions using a 2 (repeated lineups) × 2 (delay conditions) × 2 (lineup type) generalized mixed model. We also conducted additional delay conditions comparisons for Lineups 1 and 2 separately for Experiment 2 (see Additional file 1). The dependent variable was the status of choosing (choosers were coded as 1 and nonchoosers were coded as 0). Each model used the binomial link function. Participants were specified as a random effect for both models. Because we were interested in specific comparisons, we constructed a no-intercept model that produced the condition estimates as fixed effect estimates and then we performed specific comparisons (via z-tests) among the condition estimates that corresponded to the effects of interest. These multiple comparisons were conducted using the multcomp package (Hothorn, Bretz, Westfall, & Heiberger, 2012). There is no consensus on the most appropriate method for calculating degrees of freedom in mixed models. Accordingly, many R packages (including multcomp) report estimates divided by their standard errors as z values. For the sample sizes in our studies, this is a reasonable approximation. Adjusted *p*-values (Holm’s method) were reported for these multiple comparisons. Unless otherwise indicated, alpha = 0.05.

As predicted, there was a main effect of repeated lineups in both Experiment 1, *z* = − 4.16, *p* < .001, and Experiment 2, *z* = − 5.33, *p* < .001, showing that regardless of delay, the tendency to choose in Lineup 2 was greater than Lineup 1 (70.7% vs. 59.0% and 79.0% vs 67.4% in Experiments 1 and 2, respectively). In order to observe an increased tendency to choose in the subsequent lineup, most choosers must remain choosers and some nonchoosers must make a positive identification in the second lineup, suggesting that (as discussed below) the commitment and misplaced familiarity effects are likely playing a role.

Although the main effect of delay did not reach significance in Experiment 1, it was significant in Experiment 2, *z* = 3.03, *p* < .01. Participants were generally less inclined to choose after a long-initial delay (5-day delay) than a short-initial delay (10-min delay). This outcome indicates that participants were less confident in their ability to make a positive identification when the initial test was delayed. This interpretation is reflected our Welsch t-test of the higher average Lineup 1 confidence ratings of choosers in the short versus long initial delay in Experiment 1 (61.0% vs 47.3%), *t* (346.46) = 5.18, *p* < .001, and Experiment 2 (61.4% vs. 40.3%), *t* (585.74) = 9.94, *p* < .001. Moreover, because there was a main effect of repeated lineups but no interaction between repeated lineups and delay, repeated lineups seem to make participants more inclined to choose regardless of delay. Therefore, even though longer retention intervals made people less inclined to choose and lowered their confidence in their positive identifications, repeated lineups still make witnesses more inclined to choose from a subsequent lineup even with a relatively long delay.

The presence of the guilty suspect in the TP lineup did contribute to the higher TP choosing rate relative to TA choosing rate, although the effect seemed surprisingly small. The main effect of lineup type reached significance in both Experiment 1, *z* = − 2.78, *p* < .05, and Experiment 2, *z* = − 4.77, *p* < .001. However, there was also a significant interaction between delay and lineup type in Experiment 2, *z* = − 3.37, *p* < .01. Follow-up tests showed that choosing rate was greater in TP lineup than TA lineup at the 10-min delay (84.5% vs. 68.3%), *z* = − 5.39, *p* < .001, but their choosing rates were no different at a long 5-day delay (72.2% vs. 68.4%). After 5 days, participants were just as likely to choose from a TP as a TA lineup, which seems surprising because retention intervals longer than 5 days are common outside the laboratory. All other effects were not significant, *p*s > .05. The lack of an interaction between repeated lineups and lineup type indicated that the choosing rate for the second lineup increased at about the same level in both TP and TA lineups.

Together, these findings showed that both the effects of retention interval and repeated lineups influence a witness’s tendency to choose from a lineup. Participants were less inclined to choose after a long-initial delay than after a short-initial delay and even when they did choose, they were less confident. However, this effect was qualified by the presence of the guilty suspect in the TP lineup. Participants were more inclined to choose from a TP lineup than a TA lineup after a short-initial delay, but they were equally likely to choose from either lineup type after a long-initial delay (Experiment 2). Critically, regardless of delay or the lineup type, repeated lineups increased the tendency to choose. Just as in experiments in which only the suspect is repeated between two lineups, repeating all the faces in two lineups leads to an increase in choosing. We turn next to accuracy.

### Repeated identical lineups did not increase accuracy of identifying the suspect

**Fig. 4:**
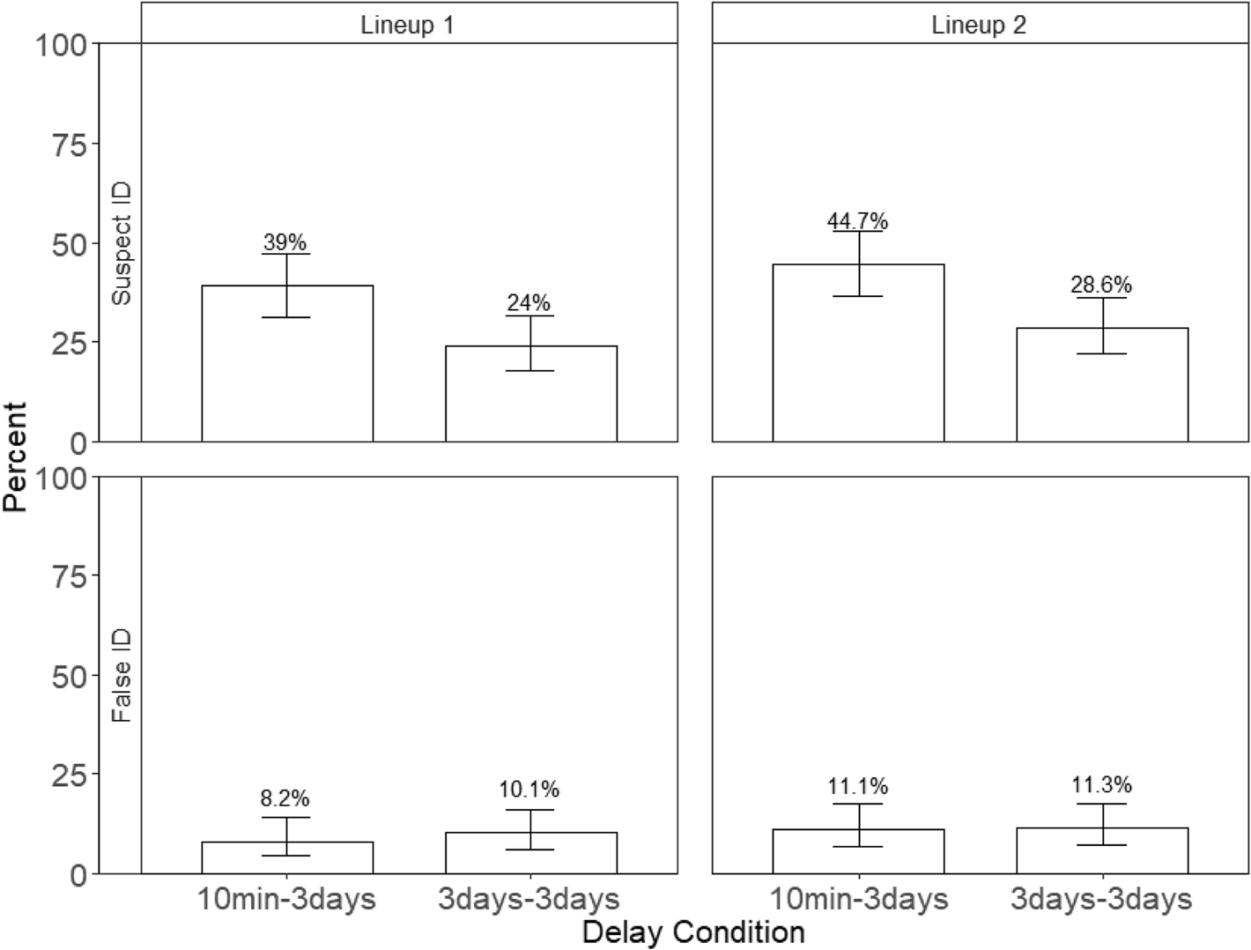
The top panels show the percentage of suspect IDs and the bottom panels show the percentage of false IDs in Experiment 1. Error bars represent 95% confidence intervals

**Fig. 5:**
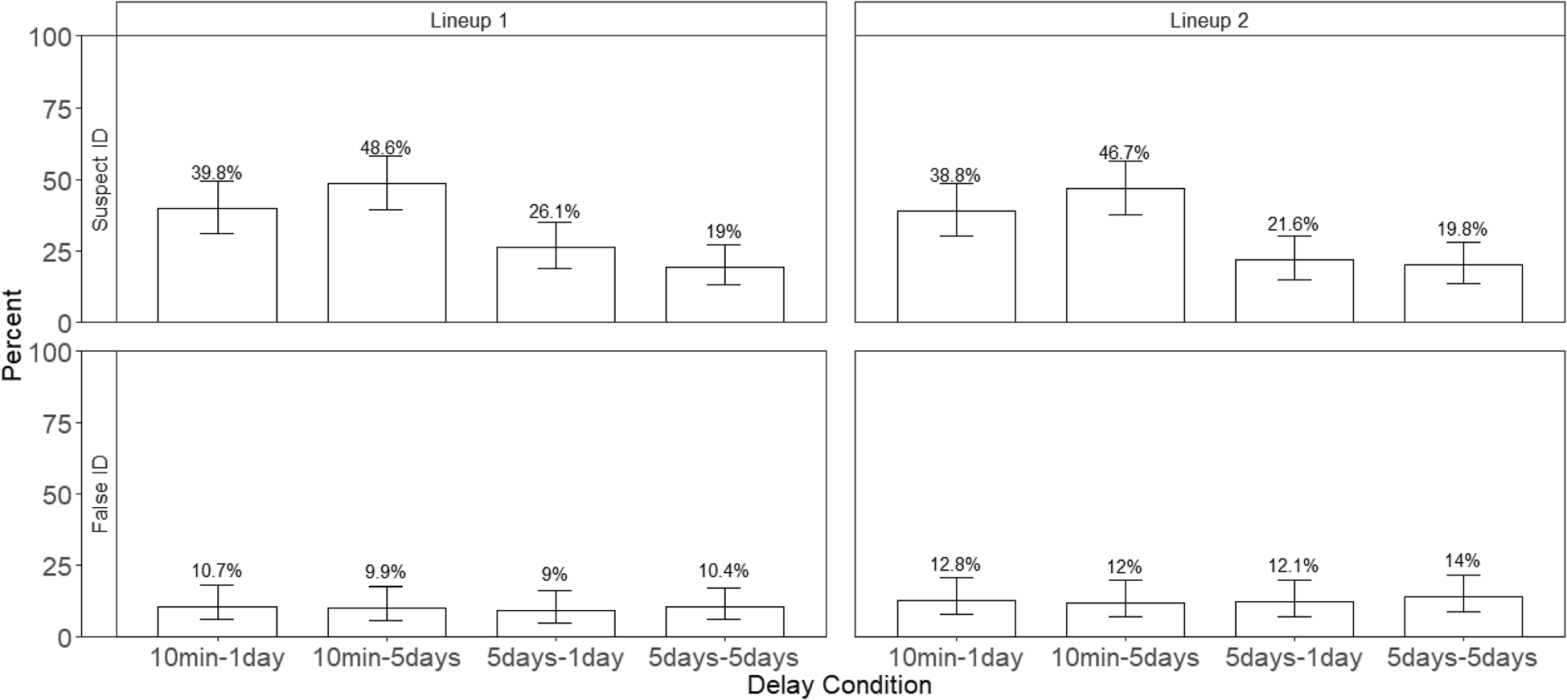
The top panels show the percentage of suspect IDs and the bottom panels show the percentage of false IDs in Experiment 2. Error bars represent 95% confidence intervals

Although participants were more likely to choose someone from a second lineup than a first lineup, they were no more accurate in picking the suspect. Accuracy is represented by the guilty suspect ID rate, which is the number of guilty suspect IDs divided by the total number of target-present lineups. Because the correct decision for a target-present lineup is a guilty suspect identification, all other possible responses in a target-present lineup (e.g., filler identification and “Not Present”) are incorrect. The top panels of Figs. 4 and 5 show the percentage of guilty suspect IDs in TP lineups in Experiments 1 and 2, respectively. We conducted a 2 (delay conditions) × 2 (repeated lineups) generalized mixed model for Experiment 1, and a 2 (delay conditions) × 2 (repeated lineups) generalized mixed model for Experiment 2. The dependent variable was accuracy (correct decisions were coded as 1 and incorrect decisions were coded as 0). The analyses here were like those conducted for choosing rates.

As predicted, the guilty suspect ID rate decreased as a function of delay in Experiment 1, *z* = 3.97, *p* < .001, and Experiment 2, *z* = 6.85, *p* < .001, but guilty suspect ID rate did not increase (or decrease) with repeated lineups, *p*s > .05 in either experiment. In other words, repeated identical lineups had no effect on overall accuracy. No hypmermnesia – improved recognition with repeated testing as obtained by Bergstein and Erdelyi (2008) in a different paradigm -- occurred. All other effects were not significant, *p*s > .05. In addition, and similar to the effects of delay on choosing rate, the average confidence when picking guilty suspects was greater on a first lineup after a short delay than after a long delay. (Experiment 1: 71.5% vs. 54.6%; Experiment 2: 72.5% vs. 48.4%). Therefore, the effects of delay were reflected in choosing rate, guilty suspect ID rate and the corresponding level of confidence when selecting guilty suspects.

### Most choosers repeated their initial decision in the second lineup identification

Although every lineup member was repeated in the second lineup, most identification decisions were carried over from the initial identification to the subsequent identification. The percentage of same and different decisions across Lineups 1 and 2 for Experiments 1 and 2 are shown in Table 1. The “same decision” category consists of identification decisions that were the same across Lineup 1 and Lineup 2 (e.g., selecting the suspect twice, a filler twice or rejecting the lineup twice). The “different decision” category consists of participants making different IDs across the lineups (e.g., selecting the suspect and then choosing a filler, among other possibilities).

**Table 1:** Experiments 1 and 2: Percentages of Same and Different Decisions across Lineup 1 and 2

Experiment	Lineup Type	Delay	Same Decision
1	Target-Present	10 min-3 days (*N* = 141)	70.9
		3 days–3 days (*N* = 154)	64.3
	Target-Absent	10 min-3 days (*N* = 143)	60.8
		3 days–3 days (*N* = 153)	64.1
2	Target-Present	10 min-1 day (*N* = 103)	70.9
		10 min-5 days (*N* = 107)	66.4
		5 days-1 day (*N* = 111)	60.4
		5 days–5 days (*N* = 121)	53.7
	Target-Absent	10 min-1 day (*N* = 109)	63.3
		10 min-5 days (*N* = 104)	54.8
		5 days-1 day (N = 109)	61.5
		5 days–5 days (*N* = 119)	54.6

Collapsing across all delay conditions, our chi-square goodness of fit test indicated a greater proportion of repeated decisions (consistent decisions) than inconsistent decisions in Experiment 1 (65.0% were consistent decisions), χ^2^[*df* = 1] = 53.01, *p* < .001, and Experiment 2 (60.5% were consistent decisions), χ^2^[*df* = 1] = 38.76, *p* < .001. Further analysis revealed that this pattern was driven primarily by choosers rather than those who rejected the lineup (nonchoosers). A 2 (choosers vs. nonchoosers) × 2 (consistent vs. inconsistent) chi-squared test confirmed this in both Experiment 1, χ^2^[*df* = 1] = 11.98, *p* < .001, and Experiment 2, χ^2^[*df* = 1] = 35.66, *p* < .001. Therefore, choosers were more likely to repeat their decisions compared to nonchoosers. This finding is consistent with choosing rate results reported in the previous section, because most choosers repeated their decisions and some nonchoosers became choosers in the subsequent lineup identification. Thus, we observed an increase in choosing rate in the subsequent lineup.

In an additional analysis, we examined whether TP and TA choosers repeated their decisions in the subsequent lineup to a similar extent. Predictably, our one-proportion z-test showed that most TP choosers remained consistent in Experiment 1 (74.7% were consistent choosers), χ^2^[*df* = 1] = 45.5, *p* < .001, and Experiment 2 (67.6% were consistent choosers), χ^2^[*df* = 1] = 40.8, *p* < .001. A similar pattern occurred with choosers in TA lineups (i.e., incorrect identifications) in both Experiment 1 (66.3% were consistent choosers), χ^2^[*df* = 1] = 17.23, *p* < .001, and Experiment 2 (67.2% were consistent choosers), χ^2^[*df* = 1] = 31.2, *p* < .001. Thus, those who erroneously picked someone in a TA lineup on a first occasion carried over the error to the second lineup nearly two-thirds of the time, confirming a commitment effect. Although identical lineups might be expected to eliminate the bias observed in situations in which a single target is repeated, we still observed a commitment effect for choosers even when all faces were familiar in the second lineups.

### Repeated lineups did not lead to confidence inflation, but there was a carryover effect

Repeated lineups could affect the CA relationship in two ways: confidence inflation and confidence carryover effect. We examined whether Lineup 2 identification decisions were made with more confidence than Lineup 1 decisions, especially for repeated decisions (consistent decisions). If Lineup 2 confidence is greater than Lineup 1 confidence, then there is a confidence inflation effect. If Lineups 1 and 2 are similar in confidence, then this outcome may indicate a confidence carryover effect.

#### Overall confidence in lineups 1 and 2

Aggregating all responses, Lineup 1 identification responses were generally made with more confidence than Lineup 2 responses in Experiment 1, *t* (590) *=* 4.78*, p* < .001, and Experiment 2, *t* (882) = 4.49, *p* < .001. Therefore, witnesses were overall more confident in Lineup 1 than Lineup 2. Next, we examined confidence ratings for consistent choosers and consistent nonchoosers (i.e., witnesses who made the same decisions in Lineups 1 and 2).

#### Consistent choosers and no-to-suspect choosers

No inflation in confidence ratings occurred for choosers who repeated their initial decisions in the second lineup (see Table 2). Consistent choosers were equally confident across lineups in Experiment 1 (56.0% to 57.3%) and Experiment 2 (54.5% to 53.0%), with Bayes factors (BF_01_) of 8.09 and 4, respectively, indicating moderate support for the null hypothesis. However, they were more confident than choosers who initially rejected but chose someone at the second lineup (no-to-suspect shift). In Experiment 1, our independent t-test indicated that witnesses who made no-to-suspect shifts in Lineup 2 were less confident than consistent choosers in Lineup 1(44.4% vs. 56.0%), *t* (350) = 3.84, *p* < .001, and at Lineup 2 (44.4% vs. 57.3%), *t* (350) = 4.14, *p* < .001. In Experiment 2, our independent t-test revealed that witnesses who made no-to-suspect shifts in Lineup 2 were again less confident than consistent choosers at Lineup 1 (43.0% vs. 54.5%), *t* (554) = 4.41, *p* < .001. Due to unequal variance in Lineup 2, we conducted a Welsch t-test and obtained the same pattern of results for our comparison of no-to-suspect shifts and consistent choosers at Lineup 2 (43.0% vs. 53.0%), *t* (309) = 4.02, *p* < .001. Therefore, although there was no overall confidence inflation for consistent choosers, they were more confident than those who initially rejected but chose someone only in the second lineup.

**Table 2:** Experiments 1 and 2: The Average Confidence Ratings for Consistent Choosers and Nonchoosers

Experiment	Choosing Status	Lineup 1	Lineup 2
1	Choosers (*N* = 247)	56.0	57.3
	Nonchoosers (*N* = 137)	62.4	53.1
2	Choosers (*N* = 401)	54.5	53.0
	Nonchoosers (*N* = 133)	57.0	48.1

#### Consistent correct and incorrect IDs

While consistent choosers were equally confident in both lineups, the level of confidence expressed did differ across response types (e.g., guilty suspect IDs vs incorrect IDs). For example, collapsing across delay conditions for Lineup 1, average confidence from consistent guilty suspect IDs in TP lineups was greater than consistent incorrect chooser IDs in TA lineups (68.0% vs. 47.6% and 66.6% vs. 51.5% in Experiments 1 and 2, respectively). The same pattern held for Lineup 2 (66.0% vs. 51.5% and 65.8% vs. 50.4%). Thus, consistent correct choosers (those who made guilty suspect IDs) were generally more confident than those who made consistent incorrect IDs in TA lineups.

#### Consistent nonchoosers

On the other hand, our paired t-test indicated that nonchoosers who repeated their decisions in the second lineup actually became significantly less confident in both Experiment 1 (62.4% to 53.1%), *t* (136) = 4.33, *p* < .001, and Experiment 2 (57.0% to 48.1%), *t* (132) = 4.62, *p* < .001. Therefore, without post-ID feedback on the first lineup, a second identical lineup did not cause confidence inflation for consistent choosers, but it made consistent nonchoosers less confident about their decisions. One possible interpretation is that the familiarity of all members of the lineup on the second occasion made it more difficult for participants to reject the lineup and thus, even when they did so, they expressed less confidence in their decision.

#### Correlation between lineups 1 and 2

Although there was no confidence inflation, Lineup 1 confidence was positively correlated with Lineup 2 confidence. In Experiment 1, we obtained a high correlation for both consistent choosers, *r* (245) = .77, *p* < .001, and for consistent nonchoosers, *r* (135) = .63, *p* < .001. In Experiment 2, we similarly found a high correlation for consistent choosers, *r* (399) = .82, *p* < .001, and for consistent nonchoosers, *r* (131) = .69, *p* < .001. Together with the average confidence results, consistent choosers and nonchoosers did not show an overall confidence inflation effect; however, their initial confidence tended to be correlated their subsequent identification confidence, suggesting the presence of a confidence carryover effect.

### Initial confidence predicted the commitment effect

Because most participants repeated their initial decisions and their initial confidence was correlated with their subsequent identification confidence, we examined whether the level of confidence expressed at Lineup 1 was predictive of repeated decisions at Lineup 2. Said differently, were witnesses who were highly confident in their first decision (correct or incorrect) more likely to provide the same decision on the second lineup than those who were less confident in their initial decisions? To evaluate this hypothesis, we categorized participants by whether they made a consistent or inconsistent decision across the two lineups. Then using the level of confidence participants expressed during Lineup 1, we calculated the proportion of these participants who repeated their decisions in Lineup 2. Specifically, we were interested in the proportion of consistent and inconsistent decisions at a high confidence cutoff (90% or above). Table 3 shows the proportion of consistent responses above and below a confidence rating of 90% in both experiments.

**Table 3:** Experiments 1 and 2: The Percentages of Consistent Responses Above and Below a Confidence Rating of 90%

Experiment	Delay	Overall		Chooser Only		Nonchooser Only	
		Above	Below	Above	Below	Above	Below
1	10 min-3 days	79.0	63.8	88.2	70.3	71.4	54.1
	3 days–3 days	73.0	63.0	81.8	68.8	69.2	52.6
	Overall	76	63.3	85.7	69.5	70.2	53.3
2	10 min-1 day	80.0	64.8	87.0	73.3	57.1	43.1
	10 min-5 days	81.5	57.6	88.9	64.1	66.7	41.5
	5 days-1 day	63.0	60.8	100	65.7	45.5	51.4
	5 days–5 days	75.0	53.1	100	58.8	57.1	42.5
	Overall	76.5	58.8	90.2	65.3	55.9	44.9

Aggregated across Experiments 1 and 2, our 2 (above vs. below 90% confidence) × 2 (consistent vs. inconsistent responses) chi-square test showed that the proportion of consistent responses above 90% confidence was greater than the proportion of consistent responses below the cutoff (76.3% vs. 60.6%, respectively), χ^2^[*df* = 1] = 14.9, *p* < .001. In addition. we decomposed overall results into data for choosers (middle columns of Table 2) and nonchoosers (right columns of Table 2). The greater consistency of among participants who rated their confidence at 90% or more held for both sets of participants, but our 2 (chooser vs. nonchooser) × 2 (consistent vs. inconsistent responses) chi-square test indicated that the effect was greater for choosers (88.6%) than for nonchoosers (64.2%), χ^2^[*df* = 1] = 13.2, *p* < .001.

In short, participants expressing high confidence were more likely to make the same choice a second time than were those expressing lower confidence on the first lineup. Thus, confidence is not only associated with identification accuracy, but initial confidence can be a predictor of the commitment effect (whether witnesses would repeat their initial decision in a subsequent lineup). Putting this outcome together with our previous findings, consistent witnesses generally maintained similar levels of confidence across repeated lineups, but their initial confidence predicted the likelihood that they would provide the same decision (right or wrong) on a subsequent identification. We also considered the effects of subsequent delay on consistent decisions; while the results were in the predicted direction with less consistent decisions after long delays, most of the analyses did not reach significance (see Additional file 1).

### A strong confidence-accuracy relationship was obtained in all conditions

The critical question for the criminal justice system is how repeated lineups and delays affect the confidence-accuracy relationship. Briefly, our results show a strong CA relationship even with repeated (identical) lineups and even with long delays. Repeated lineups did not harm the confidence-accuracy relationship in our experiments, but the procedure did not improve it either.

**Fig. 6:**
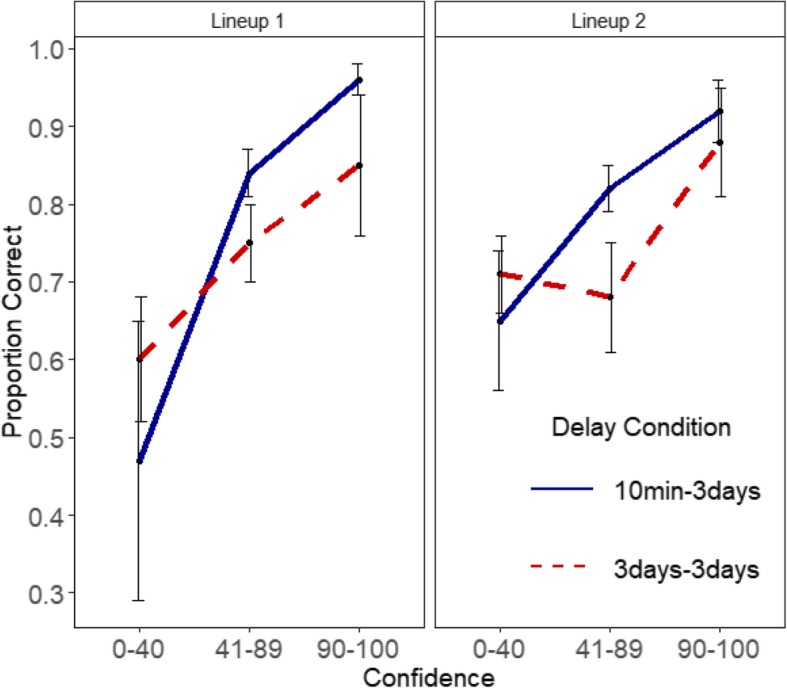
Experiment 1 CAC plots for Lineups 1 and 2. Error bars are standard errors

**Fig. 7:**
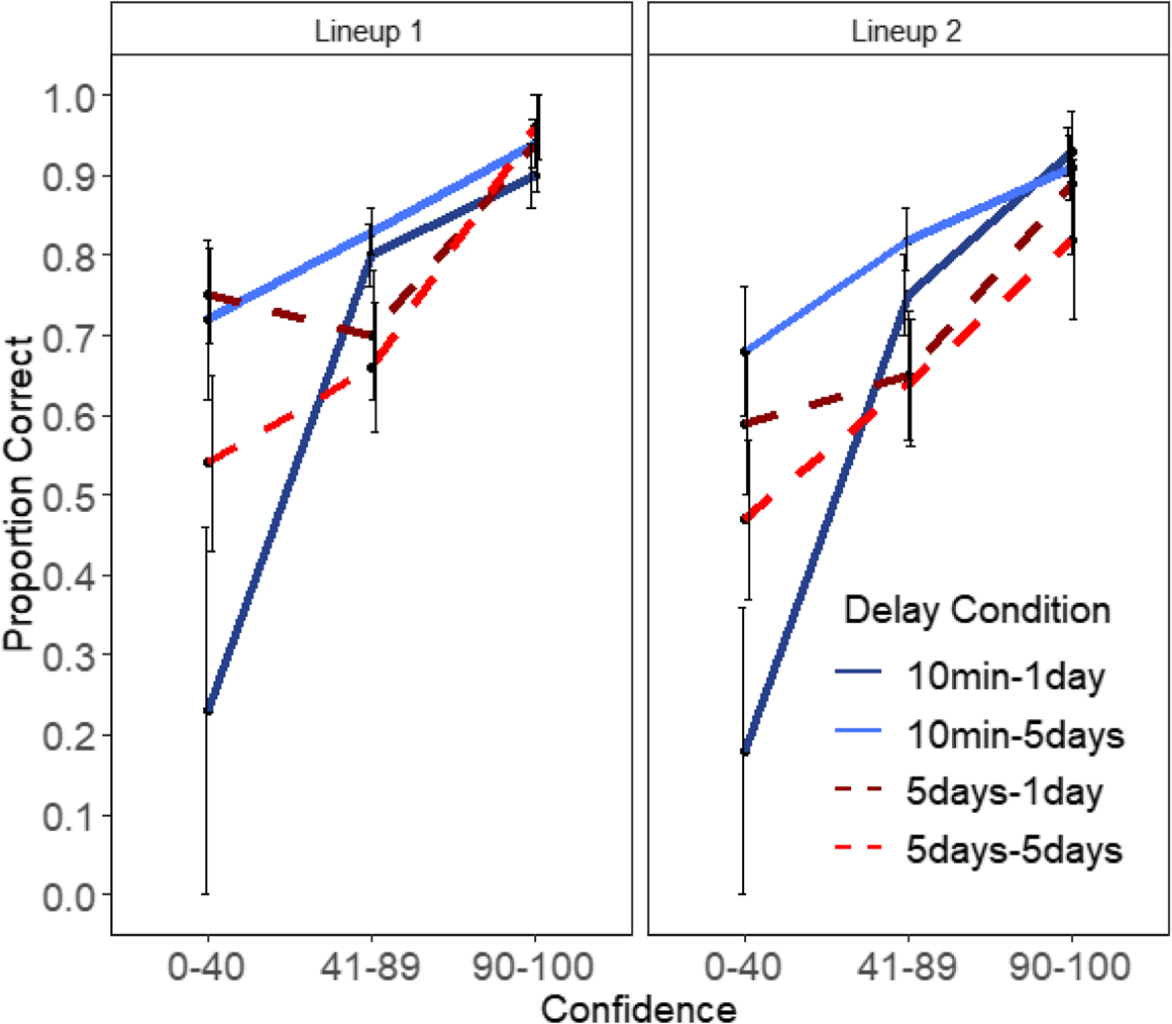
Experiment 2 CAC plots for Lineups 1 and 2. Error bars are standard errors

Figures 6 and 7 show the confidence-accuracy characteristic (CAC) plots for Lineups 1 and 2 in Experiments 1 and 2, respectively. Following the procedure from Mickes (2015) and Wixted et al. (2015) for constructing CAC plots, accuracy was calculated for each confidence bin as the number of correct suspect IDs / (correct suspects IDs + false IDs). Because there was no designated innocent suspect in the target-absent lineups, the sum of false IDs for a particular confidence bin (e.g., all the false identifications made with a confidence rating of 0–40%) was divided by 6, the lineup size (Mickes, 2015). The Additional file 1 contains the distribution of suspect and false IDs across three confidence bins (Low: 0–40%, Medium: 41–89%, and High: 90–100%). The standard error bars in the figures were computed using a jackknife procedure.

We observed a positive relationship between confidence and accuracy in both experiments (Figs. 6 and 7) even in the repeated lineup conditions and even with long delays. In Experiment 1, accuracy in the highest confidence bin for the first lineup (after 10 min) was .96 and it was .92 on the second lineup given 3 days later. When the first lineup was given after 3 days, accuracy was .86 and it was .88 on the repeated lineup 3 days later (see Fig. 6). Comparable Lineup 1 accuracy levels in Experiment 2 for the 10 min-1 day, 10 min-5 days, 5 days-1 day, and the 5 days–5 days were .90, .94, .95, and .96, respectively. The corresponding Lineup 2 accuracy levels were .93, .91, .90, and .83 (see Fig. 7). Because the error bars overlapped for the 90–100% confidence bin, there was no difference in accuracy in the various delay conditions and across the two lineups.

Consistent with prior literature (Palmer et al., 2013; Sauer et al., 2010; Wixted et al., 2016), high confidence continued to be associated with high accuracy even after a long initial delay and/or after a long subsequent delay. In addition, our study shows that this strongly positive relationship also holds true for repeated lineups. This finding, however, is not surprising considering the results in the previous sections. Most participants repeated their initial decision in the subsequent identification. Those who made a high confidence response for the first lineup were generally more likely to do so again for the second lineup. Yet despite making the same decision, they did not increase their confidence (i.e., they showed a carryover of confidence rather than its inflation). Thus, there were no significant differences in accuracy between Lineup 1 and Lineup 2 at the 90–100% confidence bin. We do not discuss confidence effects in the other bins, because only initial high confidence decisions are critical in eyewitness situations.

## General discussion

Prior research with repeated lineups (or show-ups or mugshots prior to lineups) has shown that the procedure biases selection toward the suspect, the only face repeated on the two occasions. We tried to overcome this problem by using identical lineups so that all faces were repeated, albeit in a different random order. Despite this change in procedure, our results were similar to those of prior studies that used a single repeated target (Hinz & Pezdek, 2001; Pezdek & Blandon-Gitlin, 2005; Steblay et al., 2013). When the entire lineup was repeated, and regardless of whether the suspect was in the lineup, witnesses were generally more willing to choose from a second lineup than from the initial lineup. In addition, this increase in choosing did not improve accuracy of responding. No recognition hypermnesia was obtained, as found previously (Bergstein & Erdelyi, 2008; although see Payne & Roediger, 1987). Thus, our revised procedure that eliminated the most basic flaw in all previous repeated identification procedures – only one repeated face – still did not overcome the main problems already identified in repeated lineups – familiarity and commitment effects. Besides replicating prior findings with our new procedure, we also confirmed that witnesses were less able to identify the guilty suspect after a long delay than a short delay; however, they were also less willing to make a positive identification, demonstrating their awareness of their declining retention. This interpretation is further supported by the lower confidence ratings for positive identifications after a long delay than a short delay. In addition to these findings that largely replicate past work, our results extend our understanding of repeated lineup procedures in four basic ways. We discuss these in turn and relate each finding to the prior literature.

First, we showed that, regardless of the retention interval and whether or not the lineup includes the suspect, repeated lineups exert similar effects on witnesses’ willingness to make a positive identification. When a lineup was repeated, witnesses were willing to make a positive identification despite their initial reluctance, as we observed in witnesses switching from no choice on a first lineup to choosing a suspect on the second lineup. This outcome replicated across our various initial- and subsequent-delay conditions and it is likely due to general increased familiarity of faces on the second occasion. This tendency to select a face based on familiarity counteracts the trend noted above that, on a first test, witnesses are reluctant to select a face the longer the retention interval and even when they do so, they are less confident in their choice. However, according to Godfrey and Clark (2010), repeated identification procedures may also heighten witnesses’ expectations that the suspect would be present in the lineup. Witnesses in the present study may increase choosing on the second lineup because of a shift in their decision criterion or due to misplaced familiarity, but their decision to choose was not simply due to a single-repeated target because we used identical lineups.

Second, we found that witnesses generally remain committed to their initial decision when they pick a suspect from on a repeated lineup, even when they were not biased towards choosing a single repeated target. Like studies that used a single repeated target (Memon et al., 2002; Steblay et al., 2013), we also observed the commitment and misplaced familiarity effects with identical lineups. Because all six faces were repeated in our identical lineup procedure, our results reflected the commitment and misplaced familiarity effects that could not be due to misinterpretation of the police’s (experimenter’s) intention or misplaced familiarity from a single-repeated target. Therefore, we can conclude that the misplaced familiarity and commitment effects are inherent in repeated lineup procedures even without the problem of single-face repetition. These results suggest that source monitoring (Johnson, Hashtroudi, & Lindsay, 1993) is an issue in both repeated lineups with a single-repeated target and identical lineups. When witnesses are shown a second lineup, they may misattribute their memory of faces from the first lineup for their memory of the original event. As a result, some witnesses may remain committed to their initial decision and others may choose to make a positive identification only in the second lineup. Such source monitoring errors seem ubiquitous in eyewitness research, both in eyewitness identification and from misinformation provided after witnessing a crime (Lindsay & Johnson, 1989). Our results can also be interpreted by Godfrey and Clark (2010)‘s decision criterion explanation. Witnesses who made a no-to-suspect shift at Lineup 2 were less confident than consistent choosers at Lineups 1 and 2. If we consider the average confidence expressed for positive identifications as not only a measure of witness certainty but also as an indicator of the willingness to make a positive identification based that level of certainty (i.e. decision criterion), then a lower average confidence in one group relative to another can suggest differences in decision criterion (the acceptable level of certainty to make a positive identification). It is possible that both problems in source monitoring and changing decision criteria can influence the decision process involved in repeated identifications.

Third, we showed that the initial confidence rating is a good predictor of the commitment effect. Consistent with Koriat (2012)‘s idea that confidence is a predictor of repeated judgments, we also showed that high confidence identification decisions (regardless of accuracy) are more likely to be repeated in a subsequent identification than low confidence decisions. In addition to causing the same decision, the initial level of confidence is often carried over to confidence in selecting a suspect in the subsequent lineup. Similar to Steblay et al. (2013), the consistent choosers in the present study were also equally confident across in both lineups. However, consistent nonchoosers did become less confident, which might suggest that prior familiarity can make it hard for witnesses to confidently reject a later lineup, as in other studies of misplaced familiarity (e.g., Jacoby et al., 1989). Nevertheless, both confident choosers and nonchoosers often repeat their initial decision in a subsequent identification.

Fourth, and perhaps most importantly, our experiments showed that a strong confidence-accuracy relationship remains true for repeated lineups, even ones occurring after 6-day and 10-day retention intervals since viewing the crime. Prior repeated lineup studies that reported the commitment and misplaced familiarity effects (Steblay & Dysart, 2016; Steblay et al., 2013) did not examine the influence of these effects on the confidence-accuracy relation. Our experiments lead us to conclude that the confidence-accuracy relationship remains strong despite these biasing effects. In addition, the strong relation held for all initial- and subsequent-delay conditions. Because high confidence decisions are often carried over to a subsequent lineup and the level of confidence is also often maintained (rather than inflated), repeated lineups did not significantly impair the confidence-accuracy relationship. Thus, confidence is not only predictive of accuracy across various retention interval (Palmer et al., 2013; Sauer et al., 2010), but it remains a good indicator of accuracy even for repeated lineups.

One surprise in our data is that no confidence inflation occurred on the second lineup of our repeated lineups. One great problem in the criminal justice system has been the growth in confidence over time, such that a low confidence initial judgment becomes a high confidence judgment in the courtroom. This problem is one of the root causes of people being convicted by mistaken eyewitness identification. In *Convicting the Innocent*, Brandon Garrett (2011) wrote about people convicted of crimes and then later exonerated by DNA evidence, “I expected to read that these eyewitnesses were certain at trial that they had identified the right person. They were. I did not expect, however, to read testimony by witnesses at trial indicating that they had earlier had trouble identifying the defendants … Yet in 57% of these trial transcripts (92 of 161 cases), the witnesses reported that they had *not* been certain at the time of their earlier identifications” (p. 49). He identified several causes of confidence inflation over time, such as confirmatory feedback from the police and the fact that people are often repeatedly tested. Yet in our experiments, repeated testing did not lead to confidence inflation. Our experiments are the first to use identical repeated lineups, but our results are similar to Steblay et al. (2013)’s repeated lineups with a single-repeated target. Steblay and colleagues also did not observe a confidence inflation effect. On the other hand, Godfrey and Clark (20102), Experiment found that people who consistently chose the suspect were more confident in the subsequent lineup than in the initial show-up. Therefore, confidence inflation may be possible in some circumstances, but further research is required to determine the cause. In addition, research participants and actual witnesses might approach repeated identifications differently. Compared to research participants, actual witnesses may repeatedly reenact the crime mentally over time, imagining the scene vividly and if they remember it with the wrong perpetrator as the assailant in the crime, their confidence in their judgment is likely to grow. Future research should examine the effects of repeated testing on and imagination inflation on actual witnesses.

In summary, despite our attempts to remove the biases associated with a single-repeated target, our identical lineup procedures still showed the negative effects previously reported in other repeated lineup studies. The good news is that these effects did not significantly undermine the confidence-accuracy relationship, at least in our studies with identical lineups. Therefore, when repeated lineups are not avoidable, confidence can still be a useful indicator of accuracy. Of course, this outcome needs to be replicated in the other kinds of single-target repeated lineups now being used.

## Limitations

We acknowledge there are some limitations with the present study. For example, we only used one set of lineup materials. Different eyewitness identification materials can vary in the level of difficulty, which can potentially produce different results in performance measures (e.g., choosing rate and guilty suspect ID rate). Our lineup, due to the changes we made in shirt color, was quite difficult. On the positive side, we show that even with a difficult lineup, the confidence-accuracy relation is strong: High confidence still indicates high accuracy. Thus, in this sense, the difficulty of our single lineup makes the strong CA relationship more convincing.

Another issue is that our experiments did not randomly select lineup members from a pool of faces (a filler pool), which limits the generalization of our results across different arrays of fillers. However, some of our findings, such as the commitment effect, were found in studies that had used materials different from ours. Nevertheless, we of course recommend that the effects reported in the present study be replicated using other sets of eyewitness materials.

## Implications

### Repeated lineup procedures

Steblay and Dysart (2016) had recommended against the use of repeated identification procedures involving a single repeated target; however, it was unclear from their recommendations whether repeated lineups significantly impair the confidence-accuracy relationship and whether identical lineups would be a viable alternative. The present study addressed these two issues. We showed that identical lineups still displayed the negative effects previously reported in other repeated identification studies involving a single-repeated target (Godfrey & Clark, 2010; Hinz & Pezdek, 2001; Pezdek & Blandon-Gitlin, 2005; Steblay et al., 2013). However, we also showed that the confidence-accuracy relationship was not significantly impaired by such procedure. This remained true across all of our delay manipulations. Of course, it remains to be seen if this strong relationship holds when only one face (the suspect’s) is repeated in two lineups or from a mugshot (or show up to a lineup).

Although repeated lineups did not harm the confidence-accuracy relationship, we do not argue for the general use of repeated lineups, because they have other costs. Repeated lineups led to an increased tendency to choose a suspect, regardless of delay. With only a single lineup, the greater the retention interval between witnessing the crime and seeing the lineup, the less likely witnesses are to choose a suspect and the lower their overall confidence when they do choose. However, when a lineup was repeated, people were willing to choose and this choosing sometimes occurred on the second lineup for witnesses who had declined to choose on the first lineup. Critically, repeated lineups did not improve the suspect ID rate or the confidence-accuracy relationship.

We agree with the overall recommendations from Steblay and Dysart (2016) that repeated lineups are best avoided, but it is unlikely that police will completely abandon all forms of repeated identification procedures. Thus, extra precautions should be taken to administer these procedures. As shown in the present study, using repeated lineups with the same fillers to remove the typical biases found in repeated identification procedures with a single face repeated were not enough to eliminate the negative effects. The encouraging news is that these negative effects were enough not to undermine the confidence-accuracy relationship. Therefore, confidence is still a useful indicator of witness accuracy when repeated identification is unavoidable, assuming this finding generalizes to the single repeated-lineup situation.

### Identification behaviors

Research on eyewitness identification often focuses on accurate performance, which is of course critical. Our research examined other behaviors that often receive little attention, such as the witnesses’ tendency to choose (whether right or wrong). Our experiments showed that both the length of initial delay and repeated lineups can affect the rate of choosing. People were more likely to choose after a short delay than after a long delay on a first test, and they were more likely to correctly identify the suspects and were more confident when doing so on the initial test. In addition, those who made a positive identification with high confidence were more likely to repeat their decisions and maintain their level of confidence in a subsequent lineup. Overall, witnesses were likely to choose on a second lineup than on a first lineup.

### Witness confidence

Our results add to the growing body of evidence that confidence of the witness is a powerful indicator of accuracy; the novel feature of our research is that we showed a strong confidence-accuracy relation on a second lineup as well as a first lineup (as in prior single lineup studies; Wixted et al., 2015; Wixted & Wells, 2017). In addition, we showed that this strong CA relationship exists even after a long delay (ten days) since the original viewing of the crime scenario. Of course, this conclusion may be limited to our conditions of having the same filler faces in the two lineups, a procedure not used in prior studies. Moreover, we have also shown that the confidence in the initial lineup decision also predicts the commitment effect (whether the same decision would be made when viewing the second lineup). In other words, not only does confidence predict accuracy, it also predicts the reproducibility of an identification decision.

## Conclusions

In summary, the effects of commitment and misplaced familiarity are present in both repeated lineups involving a single-repeated target and identical lineups, suggesting that these negative effects are inherent in repeated lineup procedures and were not limited to repeated identification procedures involving single-face repetition. In addition, we showed that across two experiments, repeated lineups and increasing delays contribute to people’s tendency to make a positive identification and their willingness to maintain their decisions, regardless of accuracy. Critically, however, the confidence-accuracy relationship was not significantly impaired by the negative effects of repeated lineups and delay. High confidence continued to be associated with high accuracy.

## Additional file

Additional file 1: The supplemental materials provide additional analyses of the results from our two experiments. (DOCX 2102 kb)

## Data Availability

The datasets and supplementary analyses supporting the conclusions of this article are available in the Open Science Framework repository, https://osf.io/bc3zu/.

## Abbreviations

BCa: bias-corrected and accelerated
CA: confidence-accuracy
CAC: confidence-accuracy characteristic
RT: response time or reaction time
TA: target-absent
TP: target-present
