# Supplementary material for: The effects of repeated lineups and delay on eyewitness identification
Source: Cogn Res Princ Implic. 2019 Jun 13;4:16. doi: 10.1186/s41235-019-0168-1 (PMC6565795; doi:10.1186/s41235-019-0168-1)
Supplement: Supplementary file 1 — The supplemental materials provide additional analyses of the results from our two experiments. (DOCX 2102 kb) [file 41235_2019_168_MOESM1_ESM.docx]

**Supplementary Materials**

**Lineups for Experiments 1 and 2.** The only difference between the lineups is one filler in the target present lineup, to the right of the target.

**Lineups for Experiment 1**

**
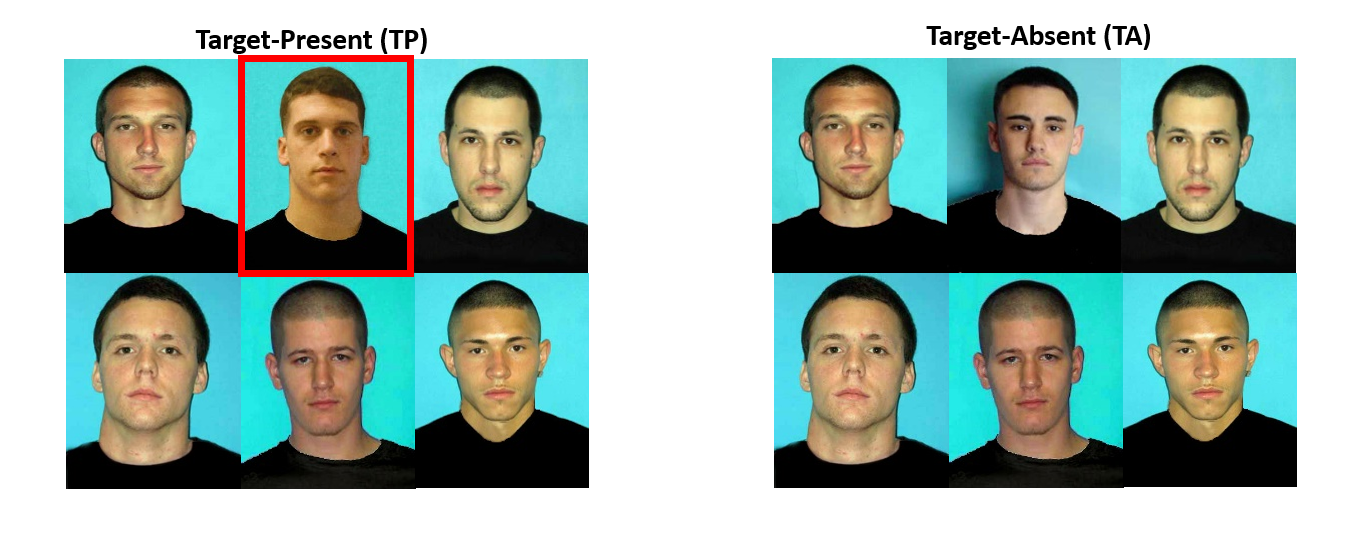
**

**Lineups for Experiment 2**

**
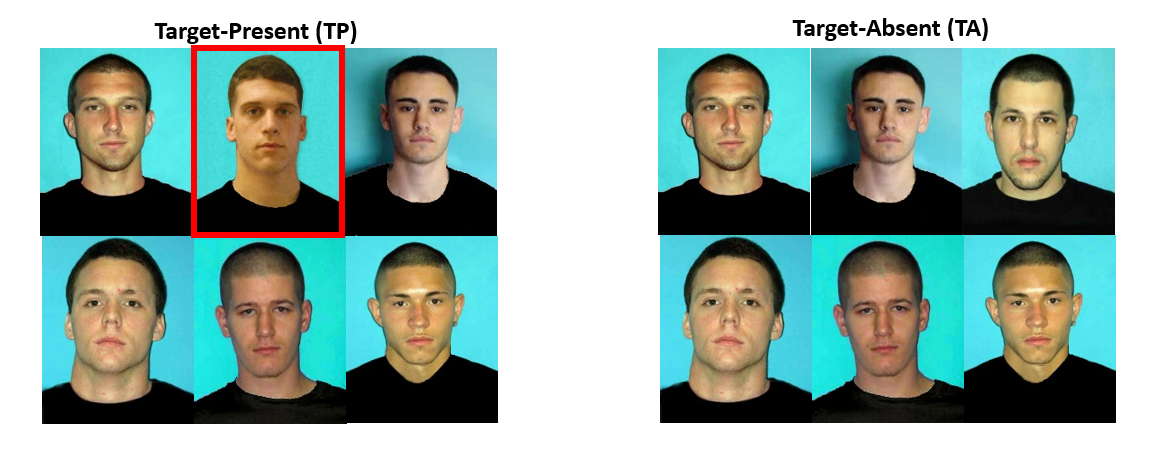
**

We use the supplemental analyses below to provide a more complete report of the results from our two experiments.

**Identification Responses in Experiment 2**

The purpose of this section is to report the comparisons of the frequency of identification responses in Experiment 2. Specifically, we compared the frequency of identification responses between the two short-initial delay conditions (10min-1day and 10min-5-days) and we also compared the frequency of identification responses between the two long-initial delay conditions (5days-1day, and 5days-5days). The data on which these analyses are based appear in Tables S1 and S2 for target-present and target-absent lineups, respectively.

Table S1
*Experiment 2: The Percentages of Identification Responses for Target-Present Lineups*

|  |  |  | Suspect | | |  | Filler | | |  | Not present | | |  | Total |  |  |  |
| --- | --- | --- | --- | --- | --- | --- | --- | --- | --- | --- | --- | --- | --- | --- | --- | --- | --- | --- |
| Lineup | Delay |  | No. | | % |  | No. | | % |  | No. | | % |  | No. |  |  |  |
| 1 | 10min-1day |  | 41 | | 39.8 |  | 43 | | 41.7 |  | 19 | | 18.4 |  | 103 |  |  |  |
|  | 10min-5days |  | 52 | | 48.6 |  | 35 | | 32.7 |  | 20 | | 18.7 |  | 107 |  |  |  |
|  | 5days-1day |  | 29 | | 26.1 |  | 51 | | 45.9 |  | 31 | | 27.9 |  | 111 |  |  |  |
|  | 5days-5days |  | 23 | | 19.0 |  | 56 | | 46.3 |  | 42 | | 34.7 |  | 121 |  |  |  |
|  | Overall |  | 145 | | 32.8 |  | 185 | | 41.9 |  | 112 | | 25.3 |  | 442 |  |  |  |
|  |  |  |  | |  |  |  | |  |  |  | |  |  |  |  |  |  |
| 2 | 10min-1day |  | 40 | | 38.8 |  | 51 | | 49.5 |  | 12 | | 11.7 |  | 103 |  |  |  |
|  | 10min-5days |  | 50 | | 46.7 |  | 43 | | 40.2 |  | 14 | | 13.1 |  | 107 |  |  |  |
|  | 5days-1day |  | 24 | | 21.6 |  | 66 | | 59.5 |  | 21 | | 18.9 |  | 111 |  |  |  |
|  | 5days-5days |  | 24 | | 19.8 |  | 62 | | 51.2 |  | 35 | | 28.9 |  | 121 |  |  |  |
|  | Overall |  | 138 | | 31.2 |  | 222 | | 50.2 |  | 82 | | 18.6 |  | 442 |  |  |  |
|  |  |  | |  |  |  | |  |  |  | |  | |  |  |  |  |  |

Table S2

|  |  | Incorrect IDs | |  | Correct Rejections | |  | Total |
| --- | --- | --- | --- | --- | --- | --- | --- | --- |
| Lineup | Delay | No. | % |  | No. | % |  | No. |
| 1 | 10min-1day | 70 | 64.2 |  | 39 | 35.8 |  | 109 |
|  | 10min-5days | 62 | 59.6 |  | 42 | 40.4 |  | 104 |
|  | 5days-1day | 59 | 54.1 |  | 50 | 45.9 |  | 109 |
|  | 5days-5days | 74 | 62.2 |  | 45 | 37.8 |  | 119 |
|  | Overall | 265 | 60.1 |  | 176 | 39.9 |  | 441 |
|  |  |  |  |  |  |  |  |  |
| 2 | 10min-1day | 84 | 77.1 |  | 25 | 22.9 |  | 109 |
|  | 10min-5days | 75 | 72.1 |  | 29 | 27.9 |  | 104 |
|  | 5days-1day | 79 | 72.5 |  | 30 | 27.5 |  | 109 |
|  | 5days-5days | 100 | 84.0 |  | 19 | 16.0 |  | 119 |
|  | Overall | 338 | 76.6 |  | 103 | 23.4 |  | 441 |

*Experiment 2: The Percentages of Identification Responses for Target-Absent Lineups*

For target-present lineups, our 3 (ID responses: suspect, filler, and not present) x 2 (delay) chi-square test of the two short-initial delay conditions (10min-1day and 10min-5-days) showed no differences in Lineup 1, *p* = .4, and Lineup 2, *p* = .4. Likewise, the two long-initial delay conditions (5days-1day, and 5days-5days) were not different from each other in Lineup 1, *p* = .3, and Lineup 2, *p* = .2. For target-absent lineups, our 2 (ID responses: false ID and not present) x 2 (delay) chi-square test of the two short-initial delay conditions (10min-1day and 10min-5-days) did not differ in Lineup 1, *p* = .5 and Lineup 2, *p* = .4. The two long-initial delay conditions (5days-1day and the 5days-5days) did not differ in Lineup 1, *p* = .2, but there was a difference in Lineup 2, (χ^2^[*df*=1] = 4.50, *p* <.05). This outcome indicates that, in target-absent lineups, the combination of a long-initial delay and a long-subsequent delay (5days-5days) resulted in more incorrect IDs in the target-absent condition than a combination of a long-initial delay and a short-subsequent delay (5days-1day). In other words, the greater proportion of incorrect IDs at Lineup 2 in the 5days-5days condition than the 5days-1day condition suggests that the longer subsequent delay led to more no-to-suspect shifts. **The Distribution of Suspect and False IDs across Confidence Bins**

The data in Table S3 show the number of responses falling into each confidence bin for subjects who identified a suspect in either target present lineups (suspect IDs) or target absent lineups (false IDs).

Table S3

*Experiments 1 and 2: The Distribution of Suspect and False IDs across Confidence Bins*

|  |  |  | Lineup 1 Confidence | | |  | Lineup 2 Confidence | | |
| --- | --- | --- | --- | --- | --- | --- | --- | --- | --- |
| Experiment | Delay | Responses | 0-40 | 41-89 | 90-100 |  | 0-40 | 41-89 | 90-100 |
| 1 | 10min-3days | Suspect IDs | 3 | 40 | 12 |  | 9 | 46 | 8 |
|  |  | False IDs | 20 | 47 | 3 |  | 29 | 62 | 4 |
|  |  |  |  |  |  |  |  |  |  |
|  | 3days-3days | Suspect IDs | 12 | 21 | 4 |  | 22 | 16 | 6 |
|  |  | False IDs | 48 | 41 | 4 |  | 53 | 46 | 5 |
|  |  |  |  |  |  |  |  |  |  |
| 2 | 10min-1day | Suspect IDs | 1 | 27 | 13 |  | 1 | 24 | 15 |
|  |  | False IDs | 20 | 41 | 9 |  | 28 | 49 | 7 |
|  |  |  |  |  |  |  |  |  |  |
|  | 10min-5days | Suspect IDs | 7 | 34 | 11 |  | 11 | 29 | 10 |
|  |  | False IDs | 16 | 42 | 4 |  | 31 | 38 | 6 |
|  |  |  |  |  |  |  |  |  |  |
|  | 5days-1day | Suspect IDs | 16 | 10 | 3 |  | 10 | 11 | 3 |
|  |  | False IDs | 32 | 26 | 1 |  | 42 | 35 | 2 |
|  |  |  |  |  |  |  |  |  |  |
|  | 5days-5days | Suspect IDs | 7 | 12 | 4 |  | 8 | 12 | 4 |
|  |  | False IDs | 36 | 37 | 1 |  | 54 | 41 | 5 |
| Notes. Because there was no designated innocent suspect, the number of false IDs should be divided by the number of lineup member, which is six. | | | | | | | | | |

The calibration accuracy in the present study was generally high, but not as high as some other calibration studies. In our case, the problem seems partly due to a particular filler whom the participants frequently mistook as the suspect. As previously mentioned, Experiments 1 and 2 had four overlapping fillers in the target-present lineup condition, but the same six fillers appeared in the target-absent lineups. Interestingly, the frequently selected filler in the TA lineup differed somewhat between Experiments 1 and 2, even though both fillers appeared in both experiments. Furthermore, a few participants did express a high confidence rating of 80% for these fillers, which suggested potential perceived similarities between these fillers and the target (see Figure S1 for the percentage of time that witnesses chose each filler in the TA lineups in the two experiments). Despite this issue, high confidence was associated with high accuracy across repeated lineups and delay conditions. More importantly, despite the combination of repeated lineups and delay, the confidence-accuracy relationship was not significantly impaired.

**
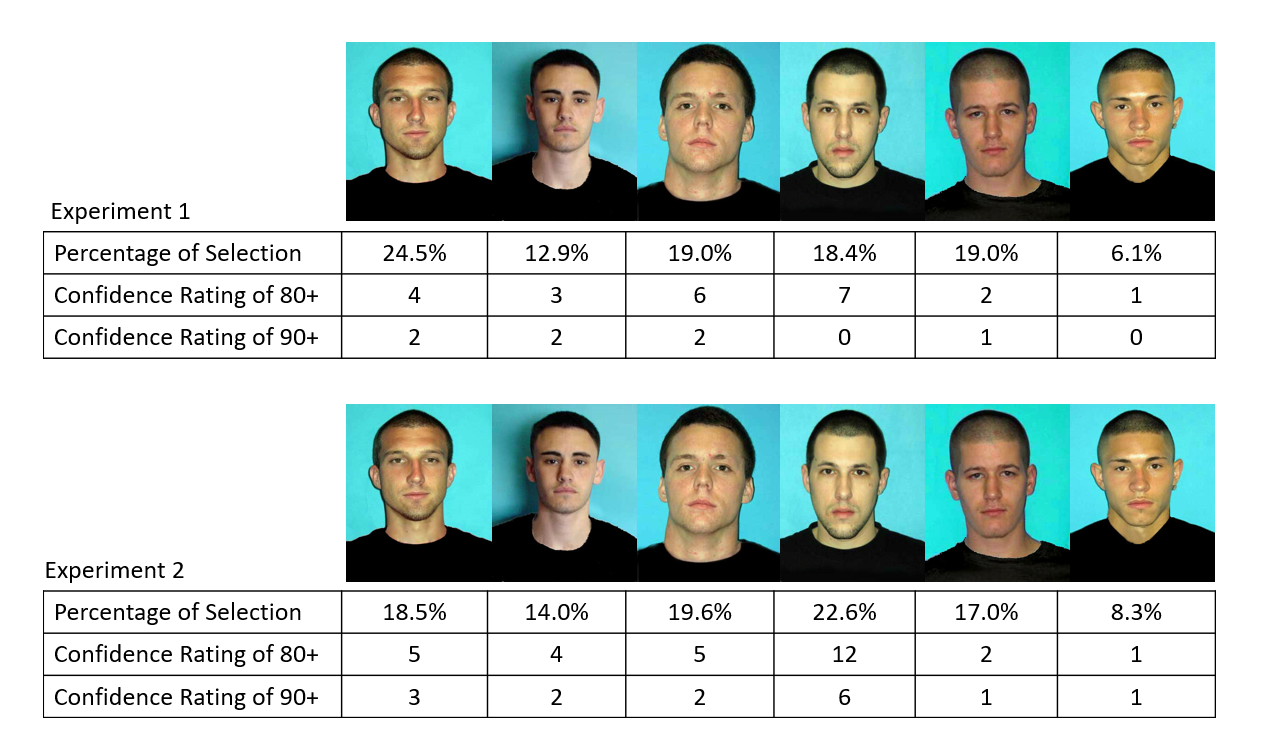
**

*Figure S1.* The percentage of target-absent filler selection and the number of target-absent filler IDs with a confidence rating of 80% or higher.

**The Subsequent Delay and Consistent Decisions**

We were interested in whether varying length of the initial delay and the subsequent delay influenced the number of consistent decisions across repeated lineups. Experiment 1 varied the length of the initial delay but not the length of the subsequent delay, whereas Experiment 2 varied in both the initial and subsequent delays. Therefore, we expected to see differences in the number of consistent decisions in Experiment 2 but not in Experiment 1. The data on which these analyses are based appear in Table 1.

**Experiment 1.** For target-present lineups, 70.9% of the participants in the 10min-3days delay condition and 64.3% of the participants in the 3days-3days delay condition maintained the same identification decision across repeated lineups. For target-absent lineups, 60.8% of the participants in the 10min-3days delay condition and 64.1% of the participants in the 3days-3days delay condition kept the same identification decision across repeated lineups. We conducted a log-linear analysis on the 2 (lineup types) x 2 (delay conditions) x 2 (consistency) frequency table. It indicated a main effect of consistency, *z* = -7.17, *p* <.001. Overall, there were generally more consistent than inconsistent responses. All other effects were not statistically significant, *ps* > .05. Importantly, the main-effects-only model provided a sufficient account of the frequencies (χ^2^[*df*=4] = 3.42, *p* = .49), indicating a clear lack of any interactions.

**Experiment 2.** We hypothesized that there would be more same decisions than different decisions when the delay between Lineups 1 and 2 was short (e.g. the 10min-1day and the 5days-1day conditions). As a consequence, there would also be more different decisions than same decisions when the delay between Lineups 1 and 2 was long (e.g. the 10min-5days and the 5days-5days conditions). Witnesses are more likely to remember their Lineup 1 decision after a short-subsequent delay than a long-subsequent delay; however, whether they repeat their Lineup 1 decision in Lineup 2 depends on 1) if they remember their Lineup 1 decision and 2) their confidence in their Lineup 1 decision. Witnesses in Lineup 1 were more confident after a short-initial delay than a long-initial delay (60.8% vs. 43.5), *t*(880) = 9.5, *p* <.001, and confident witnesses were more likely to commit to their initial decision (i.e., initial confidence predicted the same decision to be made again, or the commitment effect). Therefore, we expected that the effect of the subsequent delay would be conditional on the initial delay, because confident witnesses who remembered their initial decisions should be more likely to repeat their initial decision (e.g., those in the 10min-1day condition) compared to less confident witnesses who may have also forgotten their initial decision (e.g., those in the 5days-5days condition). Said differently, the proportion of same decisions should be higher in the 10min-1day condition (high confidence and tend to remember the decision) than the 10min-5days condition (high confidence but be more likely to forget the decision). Likewise, the proportion of same decisions should be higher in the 5days-1day condition (low confidence/remember) than the 5days-5days condition (low confidence/forget). We expected the greatest difference in the proportion of same decisions to be between the 10min-1day and the 5days-5days conditions.

We conducted a log-linear analysis of the 2 (lineup types) x 4 (delay conditions) x 2 (consistency) frequency table. The-main-effects-only model indicated an effect of consistency, *z* = -6.18, *p* <.001. Again, there were more consistent than inconsistent responses. The overall test of model adequacy indicated that the main effects provided a sufficient explanation of the table frequencies (χ ^2^[*df*=10] = 12.42, *p* = .21). However, follow-up models that included two-way interactions indicated a significant interaction between delay and consistency for target-present lineups; the 10min-1day and 5days-5days condition had significantly different numbers of consistent and inconsistent responses, *z* = 2.79, *p* <.05. 70.9% of the participants in the 10min-1day condition (when both the initial and the subsequent delay were short) made the same decisions across lineups, whereas only 53.7% of participants did so in the 5days-5days condition (when both the initial and the subsequent delay were long). All other effects were not significant, *p*s >.05.

As predicted, we did not expect to find a difference in the number of same decisions across the two delay conditions in Experiment 1with a constant subsequent delay, but we expected the data would differ in Experiment 2 with short- versus long- subsequent delays. Although the numbers of same decisions were in predicted direction, most of these differences did not reach significance. This suggest that length of the subsequent delay may only be partially responsible for the tendency to provide consistent responses across repeated lineups because most people repeated their initial decisions in the second lineup no matter how long it was delayed. In the present study, we also showed that Lineup 1 confidence is another predictor of consistency (i.e., the commitment effect). Of the two variables, Lineup 1 confidence appears to be a better predictor of consistency than the length of the subsequent delay. Considering that we did find a difference between the 10min-1day and 5days-5days condition for target-present lineups, perhaps using more varied lengths of delay would enhance its effect of finding inconsistent responses with length of the subsequent delay between lineups.

**The Utility of Confidence Judgment vs. Response Time in Determining Accuracy of Responding**

Lastly, we compared the effectiveness of confidence ratings and response times as indices of identification accuracy. To effectively use these indicators of accuracy in real-life settings, we would need to have a reliable cutoff or criterion (e.g., a confidence rating of 90% or above). Thus, the primary purpose of this section was to evaluate the utility of these indicators based on a specific cutoff. To compare the predictability of confidence ratings and response times as indicators of accuracy, we examined the number of suspect IDs and the false alarms made with high confidence (90% or above), a fast response time (10 seconds or faster) or a combination of high confidence and a fast response time. The 10 second cutoff was based on Dunning and Perretta (2002)’s claim that the optimal time-boundary that best differentiates correct choosers from incorrect choosers is 10-12 seconds. Weber and colleagues (2004) also examined their results using a 10 second cutoff. In addition, Weber et al. also examined accuracy for responses made with a confidence rating of 90% or above. For these reasons, we decided to examine our data based on these combined criteria.

The calculation of accuracy was the same as the calculation for calibration [correct suspect IDs / (correct suspects IDs + incorrect suspect IDs)]. Because there was no designated innocent suspect in the target-absent lineups, the sum of false IDs for a confidence bin (rating of 90% or above) or a RT bin (10 seconds or earlier) was divided by the number of lineup members. Accuracy was calculated for condition based on three criteria: high confidence (Confidence Only), fast response time (RT Only), and the combination of these two criteria (Confidence + RT). Table S4 show the number of responses and the accuracy based on these three criteria for Experiments 1 and 2, respectively. The “overall” row shows the aggregated the number of responses and accuracy for Lineups 1 and 2 in each of the 2 lineups in both experiments.

In both experiments, there were more fast RT responses than high confidence responses. There were, of course, also fewer responses for the combined high confidence plus fast RT criterion than the RT only and the confidence only criterion. In both Experiments 1 and 2, a high confidence criterion tended to produce higher accuracy than a fast (10 sec) response time criterion. Regardless of delay or repeated lineups, high confidence generally yielded high accuracy compared to fast response times. In some cases, accuracy was slightly improved using both the confidence and response time cutoff; however, it did not always increase accuracy. In Experiment 1, the accuracy of Lineup 1 for the 3days-3days condition was .86 with the confidence cutoff, but it was .60 with a cutoff based on both high confidence and fast response time. In other words, people who were highly confident may not always be the same people who were quick to respond. Because there were RT outliers in both Experiments 1 and 2, we examined the correlation between confidence and RT (using a continuous measure) with and without these outliers. Before any data trimming, we observed a weak correlation between confidence and RT for choosers in Experiment 2, *r*(1291) = -.11, *p* <.001, but not for choosers in Experiment 1, *r*(765) = -.04, *p* =.22. When we trimmed outliers that were longer than 1 minute, we observed a correlation for choosers in Experiment 1, *r*(749) = -.19, *p* <.001, and Experiment 2, *r*(1274) = -.21, *p* <.001. This 1-minute cutoff removed about 2.1% of the data in Experiment 1 and 1.3% of the data in Experiment 2. In short, there was a small but significant correlation between confidence and RT for choosers in both experiments. Fast choosers tended to be more accurate (if the really slow responses – more than 1 min – were eliminated.

To provide significance testing of the differences in identification accuracy across these three criteria, we computed 95% bootstrap confidence intervals via the boot package in R (Canty & Ripley, 2017). Because the bias-corrected and accelerated (BCa) method is generally recommended (Efron, 1984; Puth, Neuhäuser, & Ruxton, 2015), our 95% confidence intervals were based on this method. The reported confidence intervals were obtained from 10,000 bootstrap replications. Because there were only a few responses in some of the cells in Table S4, we aggregated the data across delay conditions before conducting the bootstrap procedure. We compared the three criteria: 1) Confidence Only vs. RT Only, 2) Confidence Only vs. Confidence + RT, and 3) RT only vs. Confidence + RT. Table S5 shows the bootstrap mean differences and their 95% BCa confidence intervals for these three comparisons. Not only was the accuracy of the confidence only criterion higher than RT only, but the 95% confidence intervals also did not include zero; therefore, the confidence only criterion was a significantly better predictor of identification accuracy than the RT criterion (10 sec or less). On the other hand, the confidence plus RT criterion and the confidence only criterion yielded similar level of accuracy. The confidence plus RT criterion generally produced higher accuracy than the RT only criterion, but these differences were relatively slight and not significant in Experiment 1. This finding suggests that at least some participants may have chosen someone quickly but were also not confident in their identification decisions. Therefore, when both fast RT and high confidence were both considered, the accuracy was similar to that of the confidence only criterion. Perhaps, more precise RTs can be measured in in-lab studies than were possible using MTurk subjects. Nonetheless, the present experiments showed that the confidence-only criterion was a more reliable measure of accuracy than relatively fast response time criterion (less than 10 secs), and that a combined measure of the two indices was not superior to the confidence-only criterion.

In sum, the present experiments showed that both the level of confidence and response times were associated with accuracy. Across both experiments, we consistently found that a high confidence criterion outperformed the fast response time criterion in determining accuracy. In contrast to Weber et al. (2004), the present study showed that the combination of post-dictive indicators (confidence + response time) was not superior to either indicator alone. The combination of both high confidence and fast response time was superior to a fast response time criterion (in Experiment 2 but not Experiment 1), but it was no better than a high confidence criterion alone (in both Experiments 1 and 2). One possible explanation for this outcome is that response time data are more variable than confidence judgments. Unlike prior response time studies (Brewer et al., 2006; Dunning & Perretta, 2002; Weber et al., 2004), the present experiments were online studies where the participants could be inattentive or distracted. Furthermore, our sample consisted of a wide age range, whereas prior studies had undergraduate participants. These factors could have contributed to the variability in our data, but of course our data also had people of various age and rather than relatively homogeneous ages of most college populations, and this difference may also have been partly responsible for the different outcomes.

Still, our results agree with those of other studies that have also produced variable response time results. For example, Weber et al. (2004) found the optimal time-boundary that differentiates correct choosers from incorrect choosers differed across all four of their studies. Likewise, the secondary analysis of our experiments also found inconsistent time-boundaries across all our conditions. Because of inconsistencies like these, it is difficult to establish a reliable response time cutoff that can differentiate correct identification decisions from incorrect ones. Confidence is the more reliable index of accuracy.

Table S4

*Experiments 1 and 2: Identification Performance Across Three Criteria of Identification Accuracy Indicators*

|  |  |  | Confidence Only | |  | RT Only | |  | Confidence + RT | |
| --- | --- | --- | --- | --- | --- | --- | --- | --- | --- | --- |
| Experiment | Lineup | Delay | N | Accuracy |  | N | Accuracy |  | N | Accuracy |
| 1 | 1 | 10min-3days | 15 | .96 |  | 61 | .88 |  | 13 | .97 |
|  |  | 3days-3days | 8 | .86 |  | 37 | .58 |  | 5 | .60 |
|  |  | Overall | 23 | .93 |  | 98 | .81 |  | 18 | .92 |
|  |  |  |  |  |  |  |  |  |  |  |
|  | 2 | 10min-3days | 12 | .92 |  | 92 | .82 |  | 9 | .92 |
|  |  | 3days-3days | 11 | .88 |  | 81 | .68 |  | 8 | .86 |
|  |  | Overall | 23 | .90 |  | 173 | .76 |  | 17 | .90 |
|  |  |  |  |  |  |  |  |  |  |  |
| 2 | 1 | 10min-1day | 22 | .90 |  | 76 | .81 |  | 19 | .89 |
|  |  | 10min-5days | 15 | .94 |  | 74 | .86 |  | 11 | .83 |
|  |  | 5days-1day | 4 | .95 |  | 41 | .71 |  | 3 | .80 |
|  |  | 5days-5days | 5 | .96 |  | 51 | .59 |  | 4 | .86 |
|  |  | Overall | 46 | .93 |  | 242 | .79 |  | 37 | .93 |
|  |  |  |  |  |  |  |  |  |  |  |
|  | 2 | 10min-1day | 22 | .93 |  | 99 | .78 |  | 20 | .95 |
|  |  | 10min-5days | 16 | .91 |  | 99 | .82 |  | 14 | .92 |
|  |  | 5days-1day | 5 | .90 |  | 76 | .67 |  | 5 | .90 |
|  |  | 5days-5days | 9 | .83 |  | 93 | .59 |  | 9 | .83 |
|  |  | Overall | 52 | .91 |  | 367 | .74 |  | 48 | .92 |

Table S5

*Experiments 1 and 2: Bootstrap Mean Differences and 95% BCa Confidence Intervals*

|  |  |  |  | 95% Confidence Interval | |
| --- | --- | --- | --- | --- | --- |
| Experiment | Lineup | Comparisons | **Δ** | Lower Bound | Upper Bound |
| 1 | 1 | Confidence vs. RT | .127 | .028 | .211 |
|  |  | Confidence vs. Confidence + RT | .009 | -.085 | .120 |
|  |  | RT vs. Confidence + RT | -.118 | -.207 | .003 |
|  |  |  |  |  |  |
|  | 2 | Confidence vs. RT | .142 | .027 | .230 |
|  |  | Confidence vs. Confidence + RT | .008 | -.116 | .152 |
|  |  | RT vs. Confidence + RT | -.134 | -.230 | .009 |
|  |  |  |  |  |  |
| 2 | 1 | Confidence vs. RT | .139 | .073 | .199 |
|  |  | Confidence vs. Confidence + RT | -.001 | -.066 | .068 |
|  |  | RT vs. Confidence + RT | -.140 | -.201 | -.068 |
|  |  |  |  |  |  |
|  | 2 | Confidence vs. RT | .168 | .095 | .229 |
|  |  | Confidence vs. Confidence + RT | -.011 | -.083 | .058 |
|  |  | RT vs. Confidence + RT | -.179 | -.240 | -.109 |

References

Canty, A., & Ripley, B. D. (2017). boot: Bootstrap R (S-Plus) Functions.

Dunning, D., & Perretta, S. (2002). Automaticity and eyewitness accuracy: a 10- to 12-second rule for distinguishing accurate from inaccurate positive identifications. *The Journal of Applied Psychology*, *87*(5), 951–962. https://doi.org/10.1037/0021-9010.87.5.951

Efron, B. (1984). Better bootstrap confidence intervals. *Journal of the American Statistical Association*, *82*(397), 171–185.

Puth, M. T., Neuhäuser, M., & Ruxton, G. D. (2015). On the variety of methods for calculating confidence intervals by bootstrapping. *Journal of Animal Ecology*, *84*(4), 892–897. https://doi.org/10.1111/1365-2656.12382

Weber, N., Brewer, N., Wells, G. L., Semmler, C., & Keast, A. (2004). Eyewitness identification accuracy and response latency: the unruly 10-12-second rule. *Journal of Experimental Psychology: Applied*, *10*(3), 139–147. https://doi.org/10.1037/1076-898X.10.3.139
